# Highly Substituted Phenol Derivatives with Nitric Oxide Inhibitory Activities from the Deep-Sea-Derived Fungus *Trichobotrys effuse* FS524

**DOI:** 10.3390/md18030134

**Published:** 2020-02-26

**Authors:** Shanchong Chen, Zhaoming Liu, Yuchan Chen, Haibo Tan, Saini Li, Hongxin Liu, Weimin Zhang, Shuang Zhu

**Affiliations:** 1 School of Biosciences and Biopharmaceutics, Guangdong Pharmaceutical University, Guangzhou 510006, China; chenshanchong@126.com; 2 State Key Laboratory of Applied Microbiology Southern China, Guangdong Provincial Key Laboratory of Microbial Culture Collection and Application, Guangdong Open Laboratory of Applied Microbiology, Guangdong Institute of Microbiology, Guangdong Academy of Sciences, Guangzhou 510070, China; liuzm@gdim.cn (Z.L.); chenyc@gdim.cn (Y.C.); maibao66@126.com (S.L.); 3 Program for Natural Products Chemical Biology, Key Laboratory of Plant Resources Conservation and Sustainable Utilization, Guangdong Provincial Key Laboratory of Applied Botany, South China Botanical Garden, Chinese Academy of Sciences, Guangzhou 510650, China; tanhaibo@scbg.ac.cn

**Keywords:** seep-sea-derived fungus, *Trichobotrys effuse*, phenol derivatives, nitric oxide inhibitory activities

## Abstract

Chemical investigation on EtOAc extract of the deep-sea-derived fungus *Trichobotrys effuse* FS524 resulted in the isolation of six new highly substituted phenol derivatives, trieffusols A–F (**1**–**6**), along with ten known relative analogs (**7**–**16**). Their structures with absolute configurations were extensively characterized on the basis of spectroscopic data analyses, single-crystal X-ray diffraction experiments, and electronic circular dichroism (ECD) calculations. Structurally, trieffusols A and B shared an unprecedented ploy-substituted 9-phenyl-hexahydroxanthone skeleton with an intriguing 6-6/6/6 tetracyclic fused ring system, which is often encountered as significant moieties in the pharmaceutical drugs but rarely discovered in natural products. In the screening towards their anti-inflammatory activities of **1**–**6**, trieffusols C and D exhibited moderate inhibitory activities against nitric oxide (NO) production in LPS-induced RAW 264.7 macrophages with IC_50_ values ranging from 51.9 to 55.9 μM.

## 1. Introduction

Marine-derived fungi have emerged as one of the most promising strategic resources to search pharmacologically significant leads for drug discovery and have aroused widespread attention from natural product chemists, pharmacologists, as well as biosynthetic chemists, due to their structurally abundant and diverse secondary metabolites in recent years [1,2]. Over the past decades, the research articles on marine natural products (MNPs) have surged dramatically, bringing about a lot of conspicuous natural products with novel chemical scaffolds and unique biological functional arrays [3,4,5,6,7]. In terms of pharmacological research, secondary metabolites derived from marine fungi are increasingly recognized as important sources of biologically meaningful natural products [8,9]. These MNPs have exhibited a wide range of biological activities such as anti-cancer [10], fungicidal [11], pro-angiogenic [12], anti-lymphangiogenic [13], and osteoclast differentiation inhibitory activities [14]. Therefore, in-depth chemical research on the MNPs would pave the way to providing potential model structures and precursor drugs for new drug developments.

**Figure 1:**
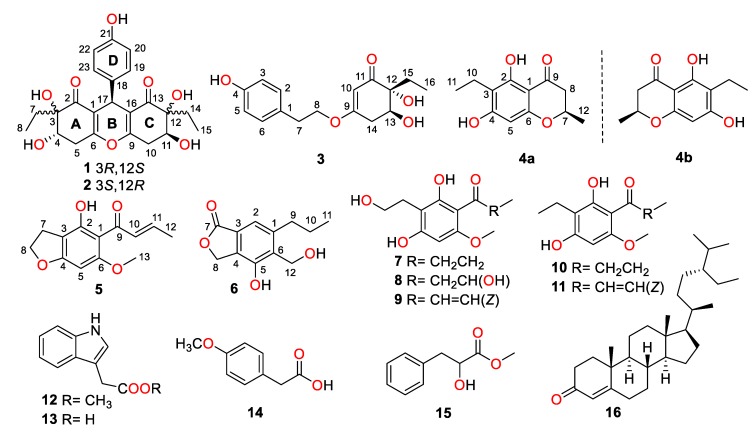
Structures of compounds **1**–**16**.

During our continuing research for structurally unique and biologically significant NPs from marine fungi [15,16,17], the fungus strain *Trichobotrys effuse* FS524, isolated from a sediment sample collecting at the South China Sea, attracted our attention, and chemical investigation of the strain resulted in the isolation of six new highly substituted phenol derivatives, trieffusols A–F (**1**–**6**), along with eleven known analogues (Figure 1), including phomalone (**7**) [18], 2,4-dihydroxy-3-(2-hydroxyethyl)-6-methoxyphenyl)-3-hydroxybutan-1-one (**8**) [18], (*E*)-1-(2,4-dihydroxy-3-(2-hydroxyethyl)-6-methoxyphenyl)but-2-en-1-one (**9**) [18], deoxyphomalone (**10**) [18], phomalichenone A (**11**) [18], methylindole-3-acetate (**12**) [19], 3-indole acetic acid (**13**) [20], 4-methoxyphenylacetic acid (**14**) [21], papuline (**15**) [22], and stigmast-4-en-3-one (**16**) [23]. Furthermore, their structures with absolute configurations were successfully established with the aid of spectroscopic data analyses, single-crystal X-ray diffraction experiments, and ECD calculations. Among them, trieffusols A and B share an intriguing 6-6/6/6 tetracyclic ring system with the formation of an unprecedented ploy-substituted 9-phenyl-hexahydroxanthone skeleton, which is often encountered as one of the most ubiquitous and intriguing functional moieties in the pharmaceutical drugs but rarely discovered in natural products. Herein, we present the isolation, structure elucidation, and biological evaluation of them in this study.

## 2. Results and Discussion

### 2.1. Structure Elucidation

Compound **1**, a colorless crystal, was given the molecular formula as C_23_H_26_O_8_ determined by the HRESIMS cationic peak at *m/z* 431.1696 [M + H]^+^ (calcd 431.1700), which corresponded to eleven degrees of unsaturation. The IR spectrum of **1** logically revealed the presence of hydroxyl and carbonyl functional groups through the characteristic resonance absorptions at 3443 cm^−1^ and 1668 cm^−1^, respectively. The further inspection of its ^1^H NMR spectrum (Table 1) clarified the existence of a *para*-substituted benzene ring resonating at [*δ*_H_ 7.11 (2H, d, *J* = 8.6 Hz, H-19, 23), 6.63 (2H, d, *J* = 8.6 Hz, H-20, 22)], three methine moieties at [*δ*_H_ 4.03 (1H, m, H-4), 4.16 (1H, m, H-11), and 4.42 (1H, s, H-17)], along with two methyl groups at [*δ*_H_ 0.77 (3H, t, *J* = 7.6 Hz, H_3_-8) and 0.83 (3H, t, *J* = 7.4 Hz, H_3_-15)]. The ^13^C NMR spectrum combined with HSQC data of **1** resolved 23 carbon resonances attributable to two methyls, four methylenes, seven methines, and ten quaternary carbons containing two keto-carbonyl ones. The aforementioned aromatic ring and functionalities logically accounted for eight degrees of unsaturation, and the remaining three degrees of unsaturation necessitated that **1** should possess an additional tricyclic ring system.

**Figure 2:**
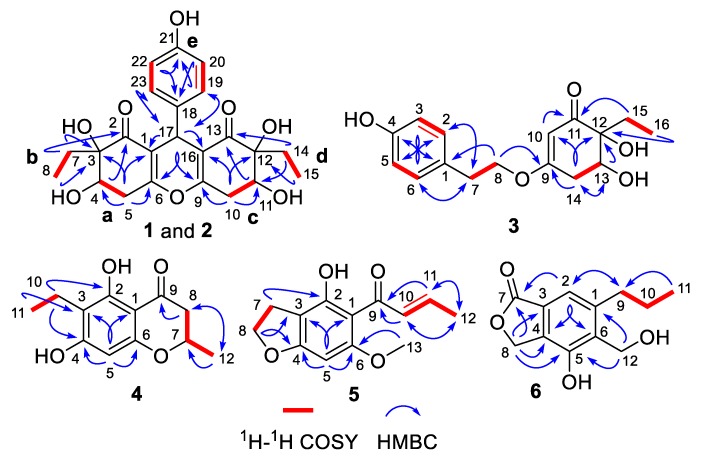
^1^H–^1^H COSY and key HMBC correlations of **1**–**6**.

The chemo-logical construction of the planar structure for compound **1** featuring a tetracyclic 6-6/6/6 ring system was elucidated by the analysis of 2D NMR spectra (Figure 2). In the ^1^H–^1^H COSY spectrum, the cross-peaks of H-4/H_2_-5, H_2_-7/H_3_-8, H_2_-10/H-11, H_2_-14/H_3_-15, and H-19/23/H-20/22 suggested the presence of five independent fragments, **a** (C-4/C-5), **b** (C-7/C-8), **c** (C-10/C-11), **d** (C-14/C-15), and **e** (C-19/23/C-20/22). The HMBC correlations from H-19/23 to C-17 and C-21, H-20/22 to C-18 and C-21, coupled with the fragment **e**, could readily confirmed the existence of the *para*-substituted benzene ring (ring D). In addition, the obvious HMBC correlations from H-4 to C-2 and C-3, H_2_-5 to C-1, C-3, C-4, and C-6, H_2_-7 to C-2 and C-3, H_3_-8 to C-3 in conjunction with fragments **a** and **b** unambiguously concluded the presence of the cyclohexenone moiety (ring A). 

Similarly, the establishment of the other cyclohexenone moiety (ring C) was confirmed by the key HMBC correlations from H_2_-10 to C-9, C-11, C-12, and C-16, H-11 to C-13, H_2_-14 to C-11 and C-13, H_3_-15 to C-12 as well as fragments **c** and **d**. Moreover, considering the remaining one degree of unsaturation and chemical shift of C-6 (*δ*_C_ 162.8) and C-9 (*δ*_C_ 161.8), we suspected that an oxygen atom should be connected between C-6 and C-9 with the formation of an oxygen bridge, which finally constructed the core pentasubstituted-4*H*-pyran skeleton (ring B). The aforementioned deduction was successfully reconfirmed by the informative HMBC correlations from H-17 to C-1, C-2, C-6, C-9, C-13, C-16, C-18, C-19, and C-23. Therefore, the planar structure of **1** was elucidated as a phenol–polyketone derivative consisting of an intriguing natural rarely-encountered 6-6/6/6 fused-ring system and given the trivial name “trieffusol A”.

**Figure 3:**
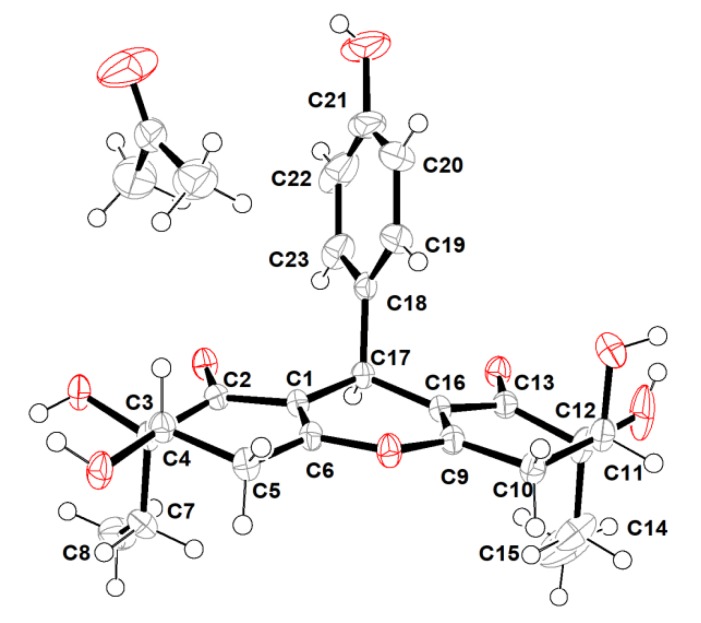
X-ray crystallographic analysis of **1**.

However, the high overlap of critical proton signals for H-4 and H-11, H_2_-7 and H_2_-14, H_2_-5, and H_2_-10, in conjunction with H_3_-8 and H_3_-15, made further construction of the relative configurations for the chiral genetic centers C-3 and C-4, as well as C-11 and C-12 in the cyclohexenone rings A and C, become a challenging issue. Moreover, all of these aforementioned carbons were far away from the central C-17 chiral genetic center, which would further give rise to two pairs of alternative diastereomeric configurations. Therefore, the assignment of the relative and absolute configurations of compound **1** through NMR and CD spectra seemed to be bleak. In order to corroborate the above structural deduction and establish absolute stereochemistry of **1**, we attempted to get X-ray crystals in the methanol/water (30:1) system. Fortunately, the single crystals with good quality were obtained and subjected to an X-ray diffraction experiment with Cu Kα radiation. The crystal data (Figure 3) not only confirmed our deduction about the planar structure of **1** but also unambiguously established its absolute configuration as 3*R*,4*S*,11*S*,12*S*,17*R*. Therefore, the complete structure with the absolute configuration of compound **1** was finally established and given the trivial name “trieffusol A”, which possesses an unprecedented ploy-substituted 9-phenyl-hexahydroxanthone skeleton with an intriguing 6-6/6/6 tetracyclic fused ring system.

**Figure 4:**
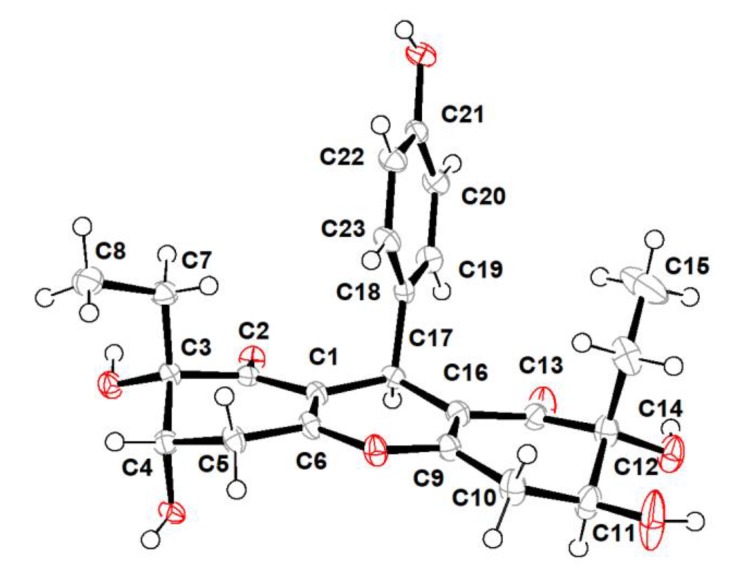
X-ray crystallographic analysis of **2**.

Trieffusol B was also obtained as a colorless crystal and had the same molecular formula C_23_H_26_O_8_ as that of **1** based on the negative mode HERSIMS (*m/z* 429.1554 [M − H]^–^, calcd 429.1555), indicating the presence of eleven degrees of hydrogen deficiency. Its ^1^H and ^13^C NMR data closely resembled those of **1**, except for chemical shift changes at C-4, C-7, C-8, C-11, C-14, and C-15. A comprehensive analysis of the 1D and 2D NMR data deduced that compounds **1** and **2** share the same planar structures, indicating that these two compounds should be a pair of diastereoisomers sharing the same ploy-substituted 9-phenyl-hexahydroxanthone skeleton with an intriguing 6-6/6/6 tetracyclic fused ring system. The aforementioned deduction was further substantiated by the X-ray single-crystallographic analysis (Figure 4), which finally clarified the absolute configuration of compound **2** as 3*S*,4*S*,11*S*,12*R*,17*S*.

Trieffusol C was purified as a brown oil. Its molecular formula was determined as C_16_H_20_O_5_ based on the protonated molecule peak at *m/z* 293.1385 [M + H]^+^ (calcd 293.1384) by HRESIMS, which requires seven degrees of unsaturation. The ^1^H NMR data of **3** (Table 2) showed various characteristic resonances responsive for a *para*-substituted aromatic ring at [*δ*_H_ 7.07 (2H, d, *J* = 8.5 Hz, H-2, 6), 6.72 (2H, d, *J* = 8.5 Hz, H-3, 5)], one trisubstituted olefinic bond at *δ*_H_ 5.34 (1H, s, H-10), one oxygenated methine moiety at *δ*_H_ 3.98 (1H, dd, *J* = 9.9, 5.9 Hz, H-13), one oxygenated methylene at *δ*_H_ 4.07 (2H, td, *J* = 6.8, 1.6 Hz, H_2_-8), together with a methyl functionality at *δ*_H_ 0.81 (3H, t, *J* = 7.5 Hz, H_3_-16). Analysis of the ^13^C NMR and HSQC data suggested the presences of 16 carbons, comprising one carbonyl carbon (*δ*_C_ 202.1), four nonprotonated carbons (*δ*_C_ 80.0, 129.7, 157.2, 176.5), five olefinic carbons (*δ*_C_ 100.8, 116.3, 116.3, 131.0, 131.0), one methine carbon (*δ*_C_ 72.6), four methylene carbons (*δ*_C_ 24.1, 35.1, 36.8, 71.5), together with one methyl carbon (*δ*_C_ 7.2).

Construction of the planar structure for **3** was accomplished by analysis of its 2D NMR data. Firstly, the presence of a *para*-substituted phenyl moiety was confirmed by the HMBC correlations from H-3/5 to C-1 and C-4, H-2/6 to C-1 and C-4, together with ^1^H-^1^H COSY correlations of H-2/6/H-3/5. Secondly, the HMBC correlations from H-10 to C-9, C-11, C-12, and C-14, H-13 to C-12 and C-15, H_2_-14 to C-9, C-10, C-12, and C-13, H_2_-15 to C-11 and C-12, H_3_-16 to C-12 and C-15, as well as ^1^H-^1^H COSY correlations of H-13/H_2_-14 and H_2_-15/H_3_-16 strongly indicated the existence of the trisubstituted cyclohex-2-en-1-one moiety. Finally, the connections of the two independent phenyl and cyclohex-2-en-1-one fragments through the linkage of C-1/C-7/C-8/O/C-9 were supported by the HMBC correlations from H-2/6 to C-7, H_2_-7 to C-1, C-2, and C-6, H_2_-8 to C-1 and C-9 as well as ^1^H-^1^H COSY fragment H_2_-7/H_2_-8. Hence, the planar structure of **3** was successfully constructed, as shown in Figure 1.

**Figure 5:**
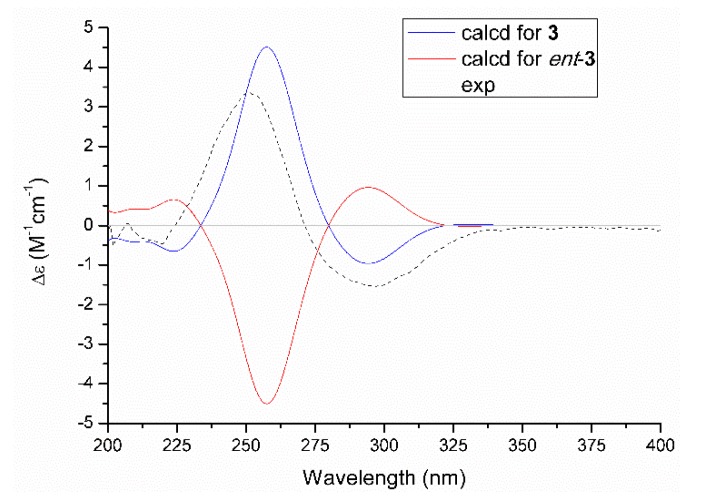
Experimental and calculated ECD spectra of **3**.

The relative configuration of **3** was assessed by the NOESY correlation, and the absence of the critical NOE correlation of H-13/H_2_-15 tentatively suggested that these two protons should orientate oppositely. The absolute stereochemistry for chiral genetic centers of C-12 and C-13 in compound **3** was determined on the basis of the comparison of experimental and the quantum mechanically calculated electric circular dichroism (ECD) data by using the time-dependent density functional theory (TDDFT) at the B3LYP/6-31+G (d,p) level in MeOH. Satisfactorily, the calculated ECD spectrum of 12*R*,13*S*-**3** (Figure 5) matched well with that of the experimental one, with a positive Cotton effect at 255 nm and a negative one at 300 nm, respectively, which unambiguously clarified the absolute configuration of **3** to be 12*R*,13*S*.

Trieffusol D was obtained as a white powder with the molecular formula of C_12_H_14_O_4,_ as deduced by HRESIMS data (*m/z* 223.0966 [M + H]^+^, calcd 223.0965). The ^1^H NMR data of **4** (Table 2) displayed one aromatic proton [*δ*_H_ 5.89 (1H, s, H-5)], one methine [*δ*_H_ 4.47 (1H, dqd, *J* = 12.3, 6.3, 3.2 Hz, H-7)], one methylene [*δ*_H_ 2.53 (2H, q, *J* = 7.4 Hz, H_2_-10)], and two methyls [*δ*_H_ 1.04 (3H, t, *J* = 7.4 Hz, H_3_-11), 1.43 (3H, d, *J* = 6.3 Hz, H_3_-12)]. Analysis of the ^13^C NMR spectrum of **4** in association with the aid of HSQC spectrum, helped to unlock 12 carbon resonances attributable to one conjugated carbonyl functional group (*δ*_C_ 198.0), six aromatic or olefinic carbons (*δ*_C_ 95.2, 103.1, 111.6, 162.4, 162.5, 165.8), one oxygenated methine (*δ*_C_ 75.2), two methylenes (*δ*_C_ 16.0, 44.2), and two methyls (*δ*_C_ 13.8, 21.0).

The ^1^H-^1^H COSY correlations of H_2_-10/H_3_-11 and the HMBC correlations from H-5 to C-1, C-3, C-4, and C-6, H_2_-10 to C-2, C-3, and C-4, H_3_-11 to C-3 and C-10 led to the establishment of the pentasubstituted benzene ring. Besides, the sequential HMBC correlations from H_2_-8 to C-7, C-9, and C-12, H_3_-12 to C-7 and C-8 along with the ^1^H-^1^H COSY correlations of H_2_-8/H-7/H_3_-12 suggested the existence of a tetrasubstituted tetrahydro-4*H*-pyran-4-one scaffold. Therefore, the gross structure of trieffusol D was established undoubtedly.

**Figure 6:**
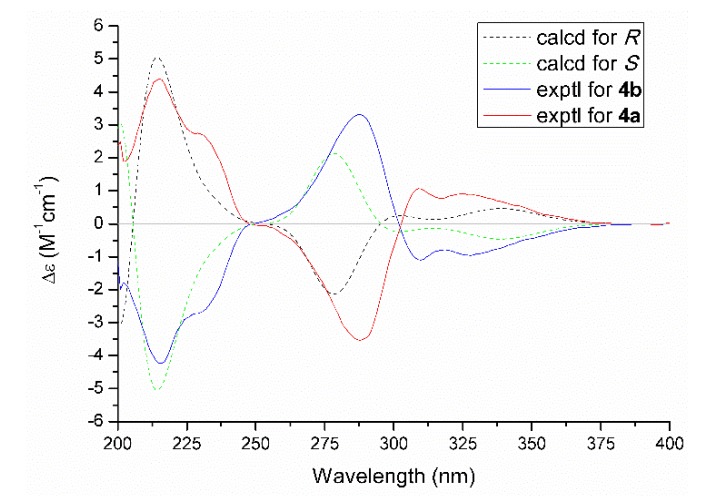
Experimental and calculated ECD spectra of **4a** and **4b**.

As for the absolute configuration, the specific optical rotation of **4** was close to zero, which logically suggested that it might exist as a pair of enantiomers. The further chiral-phase separation via chiral HPLC yielded two optically pure enantiomers **4a** and **4b**, respectively. Subsequently, the theoretical ECD spectra for **4a** and **4b** (Figure 6) were calculated by using the time-dependent density functional theory (TDDFT) at the B3LYP/6-31+G (d,p) level in MeOH. As a result, the calculated ECD spectra matched well with those of the experimental ones. Therefore, the absolute configurations of **4a** and **4b** were finally assigned as *R* and *S*, respectively.

Trieffusol E was afforded as a colorless powder and possessed a molecular formula of C_13_H_14_O_4_ based on the HRESIMS ion peak at *m/z* 235.0971 [M + H]^+^ (calcd 235.0965). Analysis of the 1D (Table 3) and 2D NMR data of **5** showed great similarity to these of 4-hydroxy-6-methoxy-5-(1′-oxobutyl)benzo[b]dihydrofuran [18]. The main difference was that the two methylenes at C-10 and C-11 positions in the known compound were oxidized to be a disubstituted double bond in **5**. This conclusion was further verified by the key HMBC correlations from H-10 to C-9 and C-12, H-11 to C-9 and C-11, H_3_-12 to C-10 and C-11, together with the ^1^H–^1^H COSY correlations of H-10/H-11/H_3_-12. Besides, the other structural identification details, as shown in Figure 2, could also support this conclusion. Moreover, the coupling constant (*J* = 15.1 Hz) between H-10 and H-11 obviously illustrated the configuration of the disubstituted double bond as an *E*-configuration. Therefore, the structure of **5** was elucidated unambiguously, as depicted in Figure 1. 

Trieffusol F was isolated as a brownish solid and determined to have a molecular formula as C_12_H_14_O_4_ from the HRESIMS data (*m/z* 223.0971 [M + H]^+^, calcd 223.0965). The ^1^H NMR data (Table 3) showed characteristic resonances for one aromatic proton [*δ*_H_ 7.21 (1H, s, H-2)], two oxygenated methylenes [*δ*_H_ 4.89 (2H, s, H_2_-12), 5.27 (2H, s, H_2_-8)], and one methyl [*δ*_H_ 1.00 (3H, t, *J* = 7.3 Hz, H_3_-11)]. The ^13^C NMR data and HSQC spectrum of **6** showed 12 carbons, which were assigned to one methyl, four methylenes (two oxygenated ones), one aromatic carbon, five quaternary carbons (one oxygenated one), and one ester carbonyl functionality. The key HMBC correlations from H-2 to C-4, C-6, and C-7, H_2_-8 to C-3, C-4, C-5, and C-7 indicated the establishment of the isobenzofuran-1(3*H*)-one moiety. Furthermore, the attachments of the C-9 at C-1 and C-12 at C-6 were confirmed by the HMBC correlations from H_2_-9 to C-1, C-2, and C-6, H-2 to C-9, H_2_-12 to C-1, C-5, and C-6, along with the ^1^H-^1^H COSY correlations of H_2_-9/H_2_-10/H_3_-11. Thus, the structure of **6** was defined, as shown in Figure 1.

### 2.2. Biological Activity

Compounds **1**–**6** were evaluated for their inhibition effect of NO production in the lipopolysaccharide (LPS)-induced mouse macrophages. As shown in Table 4, compounds **3** and **4** exhibited the inhibitory activities with IC_50_ values ranging from 51.9 to 55.9 μM, comparable to that of the positive control aminoguanidine (IC_50_: 24.8 μM). At the same time, both of them showed no cytotoxicities against macrophages, of which IC_50_ values were all greater than 200 μM.

## 3. Materials and Methods

### 3.1. General Experimental Procedures

HRESIMS data were collected on an MAT95XP machine (Thermo Fisher Scientific, Bremen, Germany). NMR spectra were acquired by an Avance-600 spectrometer (Bruker, Fällanden, Switzerland). Circular dichroism (CD) spectra were afforded by a Jasco 820 spectropolarimeter (Jasco Corporation, Kyoto, Japan). Optical rotations were obtained by an MCP-500 spectropolarimeter (Anton Paar, Graz, Austria). UV spectra were acquired using a UV-2600 spectrophotometer (Shimadzu, Kyoto, Japan). IR data were done with an Affinity-1 spectrometer (Shimadzu, Kyoto, Japan). Preparative HPLC was performed using an ODS-A column (250 × 20 mm, 5 μm, 12 nm, YMC Co., Ltd, Kyoto, Japan). An ODS-A/AQ column (250 × 10 mm, 5 μm, 12 nm, YMC CO., Ltd, Kyoto, Japan) was used for semipreparative HPLC separation and the CHIRALPAK IC column (250 × 10 mm, 5 μm) for chiral semipreparative HPLC separation. Silica gel (100–200 and 200-300 mesh, Qingdao Marine Chemical Inc., Qingdao, China), C_18_ reversed-phase silica gel (40–63 μm, Merck, Darmstadt, Germany), and Sephadex LH-20 gel (Pharmacia Fine Chemical Co. Ltd., Uppsala, Sweden) were used in the chromatography processes. Fractions were monitored by TLC, and spots were detected on heated TLC plates (silica gel GF_254_ plates, Qingdao Marine Chemical Inc., Qingdao, China) with 10% H_2_SO_4_ in EtOH under UV light.

### 3.2. Fungal Material

The strain FS524 used in this work was isolated from a sediment sample, which was collected at the depth of 1428 m in the South China Sea (110°59′04′′E, 18°00′47′′N) in June 2017. The sequence data for this strain have been submitted to the GenBank under accession no. MN545626. By using BLAST (nucleotide sequence comparison program) to search the GenBank database, FS524 has 99.8% similarity to *Trichobotrys effuse* DFFSCS021 (accession no. JX156367). The strain was preserved at the Guangdong Provincial Key Laboratory of Microbial Culture Collection and Application, Guangdong Institute of Microbiology.

### 3.3. Fermentation and Extraction

The marine fungus *T. effuse* FS524 was cultured on potato dextrose agar (PDA) at 28 °C for 7 days to prepare the seed culture, and then inoculated into flasks (3 L) each containing 9 g sea salts, 250 g of rice, and 300 mL of water. After that, all flasks were incubated at 28 °C for one month and extracted repeatedly with EtOAc. After evaporation of the solvent, a dark brown solid extract (67.3 g) was obtained. The crude extract was fractionated by silica gel column chromatography (100–200 mesh) with two gradient systems of increasing polarity (petroleum ether–EtOAc, 30:1→1:1; CH_2_Cl_2_/CH_3_OH, 10:1→1:1) to furnish nine fractions (A–I).

Fraction C (14.8 g) was subjected to silica gel CC (petroleum ether/EtOAc, 30:1→1:1) to afford seven subfractions (C1–C7). C2 was further divided into two parts (C2.1, C2.2) by Sephadex LH-20 CC (CH_2_Cl_2_–MeOH, 1:1). C2.2 was purified by semi-preparative HPLC equipped with a chiral column (isopropanol–hexane, 70:30, 2 mL/min) to yield **7** (4.1 mg, *t*_R_ = 15.8 min). C3 was separated by Sephadex LH-20 CC (CH_2_Cl_2_–MeOH, 1:1) to afford three subfractions (C3.1–C3.3). Then, semi-preparative HPLC equipped with a chiral column (isopropanol-hexane, 80:20, 2 mL/min) analysis of C3.2 afforded **9** (7.8 mg, *t*_R_ = 21.3 min) and **8** (3.4 mg, *t*_R_ = 30.1 min), respectively. Additionally, C4 was purified by semi-preparative HPLC equipped with a chiral column (isopropanol–hexane, 50:50, 2 mL/min) to obtain **5** (1.3 mg, *t*_R_ = 21.9 min) and **16** (5.4 mg, *t*_R_ = 19.5 min), respectively.

Fraction E (3.9 g) was divided into five subfractions (E1–E5) by Sephadex LH-20 CC (CH_2_Cl_2_/MeOH, 1:1). E5 was separated by semi-preparative HPLC (MeCN–H_2_O, 80:20, 2 mL/min) to yield **4** (3.1 mg, *t*_R_ = 9.2 min), **11** (4.7 mg, *t*_R_ = 11.0 min), and **10** (138.2 mg, *t*_R_ = 12.5 min). Furthermore, **4** was purified by semi-preparative HPLC equipped with a CHIRALPAK IC column (250 × 10 mm, 5 μm) (isopropanol–hexane, 20:80, 2 mL/min) to yield **4b** (1.5 mg, *t*_R_ = 12.0 min) and **4a** (1.4 mg, *t*_R_ = 14.5 min), respectively.

Fraction G (11.1 g) was subjected to C-18 reversed-phase silica gel CC (gradient elution with MeOH–H_2_O, 30:70→100:0) to afford seven subfractions (G1–G7). G1 was separated by silica gel CC (petroleum ether-EtOAc, 8:1→1:1) to afford five subfractions (G1.1–G1.5). Then semi-preparative HPLC (MeCN–H_2_O, 30:70, 2 mL/min) analysis of G1.4 afforded **3** (2.5 mg, *t*_R_ = 19.0 min). G1.5 was separated by semi-preparative HPLC (MeCN–H_2_O, 25:75, 2 mL/min) to get G1.5.2 (99.8 mg, *t*_R_ = 8.0 min) and G1.5.4 (20.5 mg, *t*_R_ = 13.8 min). G1.5.4 was further purified by semi-preparative HPLC equipped with a chiral column (isopropanol–hexane, 60:40, 2 mL/min) to yield **1** (10.8 mg, *t*_R_ = 14.4 min). Additionally, semi-preparative HPLC equipped with a chiral column (isopropanol–hexane, 45:55, 2 mL/min) analysis of G1.5.2 afforded **2** (2.3 mg, *t*_R_ = 13.2 min). G3 was divided into two subfractions (G3.1, G3.2) by Sephadex LH-20 CC (CH_2_Cl_2_–MeOH, 1:1). Then G3.1 was separated into five subfractions (G3.1.1–G3.1.5) by semi-preparative HPLC (MeCN–H_2_O, 40:60, 2 mL/min). G3.1.5 was purified by semi-preparative HPLC (MeCN–H_2_O, 45:55, 2 mL/min) and semi-preparative HPLC equipped with a chiral column (isopropanol–hexane, 60:40, 2 mL/min) to obtain **12** (8.5 mg, t_R_ = 10.0 min). Further semi-preparative HPLC equipped with a chiral column (isopropanol–hexane, 35:65, 2 mL/min) analysis of G3.1.2 and G3.1.4 afforded **13** (15.2 mg, t_R_ = 8.9 min), **14** (5.6 mg, t_R_ = 11.2 min) and **15** (19.2 mg, t_R_ = 13.6 min), respectively. G4 was divided into eight subfractions (G4.1–G4.8) by preparative HPLC (MeOH–H_2_O, 65:35, 5 mL/min). Additionally, semi-preparative HPLC equipped with a chiral column (isopropanol–hexane, 30:70, 2 mL/min) and further purified by semi-preparative HPLC (MeCN–H_2_O, 55:45, 2 mL/min) analysis of G4.5 afforded **6** (4.3 mg, t_R_ = 10.6 min).

Trieffusol A (**1**): mp 149–150 °C; colorless crystal; [*α*]${}_{D}^{25}$ +14.5 (*c* 0.12, MeOH). CD (0.35 mg/mL, MeOH): 226 (2.99), 281 (1.09), 325 (−1.38) nm. UV (MeOH) *λ*_max_ (log *ε*): 225 (4.12), 288 (3.69) nm. IR *ν*_max_: 3443, 2359, 1668, 1362, 1157 cm^−1^. ^1^H (600 MHz) and ^13^C (150 MHz) NMR spectral data, see Table 1. HRESIMS: *m*/*z* 431.1696 [M + H]^+^ (calcd for C_23_H_27_O_8_, 431.1700).

Trieffusol B (**2**): mp 148–149 °C; colorless crystal; [*α*]${}_{D}^{25}$ +33.6 (*c* 0.09, MeOH). CD (0.37 mg/mL, MeOH): 224 (2.57), 293 (0.39), 328 (−0.34) nm. UV (MeOH) *λ*_max_ (log *ε*): 224 (4.03), 293 (3.58) nm. IR data was the same as **1**. ^1^H (600 MHz) and ^13^C (150 MHz) NMR spectral data, see Table 1. HRESIMS: *m*/*z* 429.1554 [M − H]^−^ (calcd for C_23_H_25_O_8_, 429.1555).

Trieffusol C (**3**): brown oil; [*α*]${}_{D}^{25}$ +8.1 (*c* 0.12, MeOH). CD (0.39 mg/mL, MeOH): 251 (3.38), 297 (−1.53) nm. UV (MeOH) *λ*_max_ (log *ε*): 225 (3.93), 250 (3.97) nm. IR *ν*_max_: 3389, 1595, 1516, 1221, 1153, 831 cm^−1^. ^1^H (600 MHz) and ^13^C (150 MHz) NMR spectral data, see Table 2. HRESIMS: *m*/*z* 293.1385 [M + H]^+^ (calcd for C_16_H_21_O_5_, 293.1384).

(+)-Trieffusol D (**4a**): white powder; [*α*]${}_{D}^{25}$ +20.9 (*c* 0.09, MeOH). CD (0.33 mg/mL, MeOH): 215 (4.39), 288 (−3.53), 309 (1.07) nm. UV (MeOH) *λ*_max_ (log *ε*): 213 (4.03), 292 (3.90) nm. IR *ν*_max_: 2930, 1585, 1306, 1119, 716 cm^−1^. ^1^H (600 MHz) and ^13^C (150 MHz) NMR spectral data, see Table 2. HRESIMS: *m*/*z* 223.0966 [M + H]^+^ (calcd for C_12_H_15_O_4_, 223.0965).

(−)-Trieffusol D (**4b**): white powder; [*α*]${}_{D}^{25}$ −24.7 (*c* 0.07, MeOH). CD (0.34 mg/mL, MeOH): 216 (−4.24), 287 (3.31), 310 (−1.10) nm. UV (MeOH) *λ*_max_ (log *ε*): 213 (3.98), 292 (3.88) nm. IR data was the same as **4a**. ^1^H (600 MHz) and ^13^C (150 MHz) NMR spectral data, see Table 2. HRESIMS: *m*/*z* 221.0823 [M − H]^−^ (calcd for C_12_H_13_O_4_, 221.0819).

Trieffusol E (**5**): colorless powder; UV (MeOH) *λ*_max_ (log *ε*): 207 (3.88), 241 (3.59), 324 (3.62) nm. IR *ν*_max_: 3362, 1418, 1020, 642 cm^−1^. ^1^H (600 MHz) and ^13^C (150 MHz) NMR spectral data, see Table 3. HRESIMS: *m*/*z* 235.0971 [M + H]^+^ (calcd for C_13_H_15_O_4_, 235.0965).

Trieffusol F (**6**): brownish solid; UV (MeOH) *λ*_max_ (log *ε*): 208 (3.74), 250 (3.19), 301 (2.81) nm. IR *ν*_max_: 3310, 1748, 1522, 1009, 773 cm^−1^. ^1^H (600 MHz) and ^13^C (150 MHz) NMR spectral data, see Table 3. HRESIMS: *m*/*z* 223.0971 [M + H]^+^ (calcd for C_12_H_15_O_4_, 223.0965).

### 3.4. X-ray Crytallographic Data of Compounds ***1*** and ***2***

The single-crystal X-ray diffraction data for compounds **1** and **2** were collected on an Agilent Xcalibur Nova single-crystal diffractometer using CuK*α* radiation at 293 and 100 K, respectively. The crystal structures were refined by full-matrix least-squares calculation (for details see X-ray crystallographic analysis, Tables S1 and S2 in the supporting information). Crystallographic data have been deposited at the Cambridge Crystallographic Data Center with the deposition number of CDCC 1974673 for **1** and CDCC 1974674 for **2**, respectively. Copies of these data can be obtained free of charge via www.ccdc.cam.au.ck/conts/retrieving.html.

### 3.5. Quantum Chemical Calculations

Merck molecular force field (MMFF) and DFT/TD-DFT calculations were carried out with the Spartan’14 software (Wavefunction Inc., Irvine, CA, USA) and the Gaussian 09 program, respectively [24]. Conformers within the 10 kcal mol^−1^ energy window were generated and optimized using DFT calculations at the B3LYP/6-31+G (d,p) level. Frequency calculations were performed at the same level to confirm that each optimized conformer was true minimum and to estimate their relative thermal free energy (ΔG) at 298.15 K. Conformers with the Boltzmann distribution over 5% were chosen for ECD calculations in methanol at the B3LYP/6-311+G (d,p) level. Solvent effects were taken into consideration using the self-consistent reaction field (SCRF) method with the polarizable continuum model (PCM) [25]. Details of the individual conformers are provided in the supporting information. The ECD spectrum was generated by the SpecDis program [26] using a Gaussian band shape with 0.26 eV exponential half-width from dipole-length dipolar and rotational strengths.

### 3.6. Nitric Oxide Inhibitory Activities Assay

Compounds **1**–**6** were evaluated for the inhibitory activity of nitric oxide (NO) production in lipopolysaccharide (LPS)-induced RAW 246.7 mouse macrophages [27]. The cells (180 μL) with a density of 5 × 10^5^ cells/mL of media on a 96-well plate were put under 37 °C at a 5% CO_2_ condition. After a 24 h preincubation, the seeded cells were treated with gradient dilutions of **1**-**6** with a maximum concentration of 100 μM, followed by stimulation with LPS (1 μg/mL) for 24 h. Then 50 μL cell culture supernatant solution was moved to a new plate that contained NO detection Griess A (50 μL) and Griess B (50 μL). Finally, the absorbance was measured at 540 nm. Aminoguanidine was used as a positive control and all data were obtained in triplicate. The viability of RAW264.7 cells was evaluated according to the SRB method simultaneously to exclude the interference of the cytotoxicity of **1**–**6**. The RAW264.7 cells were purchased from the Cell Bank of the Chinese Academy of Sciences.

## 4. Conclusions

In summary, six new highly substituted phenol derivatives, trieffusols A–F (**1**–**6**), along with eleven known analogs (**7**–**16**), were identified from the deep-sea-derived fungus *Trichobotrys effuse* FS524. Interestingly, trieffusols A and B share an intriguing 6-6/6/6 tetracyclic ring system with the formation of an unprecedented ploy-substituted 9-phenyl-hexahydroxanthone skeleton, which is often encountered as one of the most ubiquitous and intriguing functional moieties in the pharmaceutical drugs but rarely discovered in natural products. All the compounds were screened for their nitric oxide (NO) inhibitory activities, compounds **3** and **4** exhibited moderate inhibitory activities against NO production in LPS-induced RAW 264.7 macrophages.

## Figures and Tables

**Table 1:** ^1^H (600 MHz) and ^13^C (150 MHz) NMR data for compounds **1** and **2** in CD_3_COCD_3_.

No.	1		2	
	*δ*_H_ (*J* in Hz)	*δ*_C_	*δ*_H_ (*J* in Hz)	*δ*_C_
1		115.2, C		113.6, C
2		198.8, C		199.8, C
3		79.2, C		79.6, C
4	4.03 (m)	71.3, CH	4.13 (m)	72.3, CH
5*α* 5*β*	2.63 (dd, 18.1, 8.7) 3.00 (dd, 18.1, 5.7)	33.9, CH_2_	2.63 (dd, 18.5, 1.9) 3.20 (dd, 18.5, 4.1)	33.7, CH_2_
6		162.8, C		158.9, C
7a 7b	1.56 (m) 1.92 (dq, 15.0, 7.6)	23.5, CH_2_	1.50 (m)	28.8, CH_2_
8	0.77 (t, 7.6)	7.0, CH_3_	0.32 (t, 7.4)	6.9, CH_3_
9		161.8, C		160.3, C
10*α* 10*β*	3.06 (dd, 18.8, 3.9) 2.83 (dd, 18.8, 2.6)	33.5, CH_2_	2.83 (m) 2.75 (dd, 17.6, 9.9)	34.3, CH_2_
11	4.16 (m)	70.9, CH	4.09 (m)	72.2, CH
12		79.3, C		80.1, C
13		199.2, C		199.7, C
14a 14b	1.63 (m)	29.4, CH_2_	1.45 (m) 1.81 (m)	23.3, CH_2_
15	0.83 (t, 7.4)	7.8, CH_3_	0.20 (t, 7.5)	6.2, CH_3_
16		113.5, C		114.3, C
17	4.42 (s)	32.8, CH	4.70 (s)	32.8, CH
18		136.2, C		135.6, C
19	7.11 (d, 8.6)	130.2, CH	7.07 (d, 8.5)	130.5, CH
20	6.63 (d, 8.6)	115.4, CH	6.66 (d, 8.5)	115.4, CH
21		156.7, C		156.9, C
22	6.63 (d, 8.6)	115.4, CH	6.66 (d, 8.5)	115.4, CH
23	7.11 (d, 8.6)	130.2, CH	7.07 (d, 8.5)	130.5, CH
21-OH	8.09 (s)		8.14 (s)	

**Table 2:** ^1^H (600 MHz) and ^13^C (150 MHz) NMR data for compounds **3** and **4** in CD_3_OD.

No.	3		4	
	*δ*_H_ (*J* in Hz)	*δ*_C_	*δ*_H_ (*J* in Hz)	*δ*_C_
1		129.7, C		103.1, C
2	7.07 (d, 8.5)	131.0, CH		162.4, C
3	6.72 (d, 8.5)	116.3, CH		111.6, C
4		157.2, C		165.8, C
5	6.72 (d, 8.5)	116.3, CH	5.89 (s)	95.2, CH
6	7.07 (d, 8.5)	131.0, CH		162.5, C
7	2.94 (t, 6.8)	35.1, CH_2_	4.47 (dqd, 12.3, 6.3, 3.2)	75.2, CH
8	4.07 (td, 6.8, 1.6)	71.5, CH_2_	2.58 (d, 3.2) 2.65 (17.1, 12.3)	44.2, CH_2_
9		176.5, C		198.0, C
10	5.34 (s)	100.8, CH	2.53 (q, 7.4)	16.0, CH_2_
11		202.1, C	1.04 (t, 7.4)	13.8, CH_3_
12		80.0, C	1.43 (d, 6.3)	21.0, CH_3_
13	3.98 (dd, 9.9, 5.9)	72.6, CH		
14a	2.53 (dd, 17.7, 9.9)	36.8, CH_2_		
14b	2.68 (dq, 17.7, 5.9)			
15a	1.58 (dq, 14.3, 7.5)	24.1, CH_2_		
15b	1.89 (dd, 14.3, 7.5)			
16	0.81 (t, 7.5)	7.2, CH_3_		

**Table 3:** ^1^H (600 MHz) and ^13^C (150 MHz) NMR data for compounds **5** and **6** in CD_3_OD.

No.	5		6	
	*δ*_H_ (*J* in Hz)	*δ*_C_	*δ*_H_ (*J* in Hz)	*δ*_C_
1		106.8, C		145.5, C
2		163.0, C	7.21 (s)	117.7, CH
3		106.5, C		126.7, C
4		169.1, C		132.7, C
5	6.07 (s)	87.2, CH		152.6, C
6		165.5, C		131.6, C
7	3.07 (t, 8.7)	26.7, CH_2_		173.8, C
8	4.66 (t, 8.7)	74.4, CH_2_	5.27 (s)	69.7, CH_2_
9		194.6, C	2.73 (m)	36.0, CH_2_
10	7.21 (dd, 15.1, 1.6)	133.5, CH	1.64 (m)	25.5, CH_2_
11	6.98 (dq, 15.1, 6.9)	142.9, CH	1.00 (t, 7.3)	14.3, CH_3_
12	1.94 (dd, 6.9, 1.6)	18.6, CH_3_	4.89 (s)	58.2, CH_2_
13	3.86 (s)	56.4, CH_3_		

**Table 4:** Inhibitory effects of **1**-**6** on the NO production and the cytotoxicity.

Compounds	Inhibition of NO Production (IC_50_/μM) ^a^	Cytotoxicity (IC_50_/μM)
**1**	>200	>200
**2**	108.1 ± 3.0	>200
**3**	51.9 ± 1.4	>200
**4a**	54.3 ± 2.2	>200
**4b**	55.9 ± 1.6	>200
**5**	65.5 ± 1.3	>200
**6**	111.2 ± 4.6	>200
Aminoguanidine	24.8 ± 0.8	>200

^a^ Values are expressed as the mean ± SD.

## Supplementary Materials

The following are available online at https://www.mdpi.com/1660-3397/18/3/134/s1. Figures S1–S2: B3LYP/6-31+G (d,p) optimized low-energy conformers of **3** and **4**; Figures S3–S58: 1D, 2D NMR, HRESIMS, CD, UV and IR spectra of compounds **1**–**6**.
