# Supplementary material for: Highly Substituted Phenol Derivatives with Nitric Oxide Inhibitory Activities from the Deep-Sea-Derived Fungus Trichobotrys effuse FS524
Source: Mar Drugs. 2020 Feb 26;18(3):134. doi: 10.3390/md18030134 (PMC7143758; doi:10.3390/md18030134)
Supplement: Supplementary file 1 [file marinedrugs-18-00134-s001.zip › Supplementary Files/Supporting Information.pdf]

Supporting Information

Highly Substituted Phenol Derivatives with Nitric Oxide Inhibitory Activities from the Deep-Sea-Derived Fungus *Trichobotrys effuse* FS524

Shanchong Chen[1,2], Zhaoming Liu[2], Yuchan Chen[2], Haibo Tan[3], Saini Li[2], Hongxin Liu*[,2], Weimin Zhang*[,2], Shuang Zhu*[,1]

[1]School of Biosciences and Biopharmaceutics, Guangdong Pharmaceutical University, Guangzhou 510006, China

[2]State Key Laboratory of Applied Microbiology Southern China, Guangdong Provincial Key Laboratory of Microbial Culture Collection and Application, Guangdong Open Laboratory of Applied Microbiology, Guangdong Institute of Microbiology, Guangdong Academy of Sciences, Guangzhou 510070, China

[3]Program for Natural Products Chemical Biology, Key Laboratory of Plant Resources Conservation and Sustainable Utilization, Guangdong Provincial Key Laboratory of Applied Botany, South China Botanical Garden, Chinese Academy of Sciences, Guangzhou 510650, China

Contents

1. Experimental Section

1.1 X-ray crystallographic analysis of compounds **1** and **2**.

1.2 Computational data

2. NMR, HRESIMS, CD, UV and IR spectra of compounds 1-6

Figure S3. ^{1}H NMR spectrum (600 MHz, CD_3COCD_3) of **1**

Figure S4. ^{13}C NMR spectrum (150 MHz, CD_3COCD_3) of **1**

Figure S5. ^{1}H-^{1}H COSY spectrum of **1** in CD_3COCD_3

Figure S6. HSQC spectrum of **1** in CD_3COCD_3

Figure S7. HMBC spectrum of **1** in CD_3COCD_3

Figure S8. NOESY spectrum of **1** in CD_3COCD_3

Figure S9. HRESIMS spectrum of **1**

Figure S10. CD spectrum of **1**

Figure S11. UV spectrum of **1**

Figure S12. IR spectrum of **1**

Figure S13. ^{1}H NMR spectrum (600 MHz, CD_3COCD_3) of **2**

Figure S14. ^{13}C NMR spectrum (150 MHz, CD_3COCD_3) of **2**

Figure S15. ^{1}H-^{1}H COSY spectrum of **2** in CD_3COCD_3

Figure S16. HSQC spectrum of **2** in CD_3COCD_3

Figure S17. HMBC spectrum of **2** in CD_3COCD_3

Figure S18. NOESY spectrum of **2** in CD_3COCD_3

Figure S19. HRESIMS spectrum of **2**

Figure S20. CD spectrum of **2**

Figure S21. UV spectrum of **2**

Figure S22. ^{1}H NMR spectrum (600 MHz, CD_3OD) of **3**

Figure S23. ^{13}C NMR spectrum (150 MHz, CD_3OD) of **3**

Figure S24. ^{1}H-^{1}H COSY spectrum of **3** in CD_3OD

Figure S25. HSQC spectrum of **3** in CD_3OD

Figure S26. HMBC spectrum of **3** in CD_3OD

Figure S27. NOESY spectrum of **3** in CD_3OD

Figure S28. HRESIMS spectrum of **3**

Figure S29. CD spectrum of **3**

Figure S30. UV spectrum of **3**

Figure S31. IR spectrum of **3**

Figure S32. ^1H NMR spectrum (600 MHz, CD_3OD) of **4**

Figure S33. ^{13}C NMR spectrum (150 MHz, CD_3OD) of **4**

Figure S34. ^1H-^1H COSY spectrum of **4** in CD_3OD

Figure S35. HSQC spectrum of **4** in CD_3OD

Figure S36. HMBC spectrum of **4** in CD_3OD

Figure S37. HRESIMS spectrum of **4a**

Figure S38. HRESIMS spectrum of **4b**

Figure S39. CD spectrum of **4a**

Figure S40. CD spectrum of **4b**

Figure S41. UV spectrum of **4**

Figure S42. IR spectrum of **4**

Figure S43. ^1H NMR spectrum (600 MHz, CD_3OD) of **5**

Figure S44. ^{13}C NMR spectrum (150 MHz, CD_3OD) of **5**

Figure S45. ^1H-^1H COSY spectrum of **5** in CD_3OD

Figure S46. HSQC spectrum of **5** in CD_3OD

Figure S47. HMBC spectrum of **5** in CD_3OD

Figure S48. HRESIMS spectrum of **5**

Figure S49. UV spectrum of **5**

Figure S50. IR spectrum of **5**

Figure S51. ^1H NMR spectrum (600 MHz, CD_3OD) of **6**

Figure S52. ^{13}C NMR spectrum (150 MHz, CD_3OD) of **6**

Figure S53. ^1H-^1H COSY spectrum of **6** in CD_3OD

Figure S54. HSQC spectrum of **6** in CD_3OD

Figure S55. HMBC spectrum of **6** in CD_3OD

Figure S56. HRESIMS spectrum of **6**

Figure S57. UV spectrum of **6**

Figure S58. IR spectrum of **6**

1.1 X-ray crystallographic analysis of compounds **1** and **2**.

Table S1. X-ray crystallographic data for **1**.

Empirical formula	$C_{29}H_{38}O_{10}$
Formula weight	546.59
Temperature/K	293(2)
Crystal system	monoclinic
Space group	$P2_1$
a/Å	12.70640(10)
b/Å	7.79060(10)
c/Å	14.07730(10)
α/°	90
β/°	99.0070(10)
γ/°	90
Volume/Å^3	1376.34(2)
Z	2
ρ_{calc} g/cm^3	1.319
μ/mm^{-1}	0.824
F(000)	584.0
Crystal size/mm^3	0.2 × 0.18 × 0.10
Radiation	CuKα (λ = 1.54184)
2Θ range for data collection/°	8.722 to 148.848
Index ranges	$-15 \leq h \leq 15$, $-9 \leq k \leq 9$, $-15 \leq l \leq 17$
Reflections collected	13950
Independent reflections	5450 [R_{int} = 0.0199, R_{sigma} = 0.0175]
Data/restraints/parameters	5450/1/363
Goodness-of-fit on F^2	1.031
Final R indexes [I>=2σ (I)]	R_1 = 0.0443, wR_2 = 0.1210
Final R indexes [all data]	R_1 = 0.0445, wR_2 = 0.1213
Largest diff. peak/hole / e Å^{-3}	0.41/-0.49
Flack parameter	-0.02(4)

Table S2. X-ray crystallographic data for **2**.

Empirical formula	$C_{46}H_{54}O_{17}$
Formula weight	878.89
Temperature/K	100(1)
Crystal system	monoclinic
Space group	$P2_1$
a/Å	15.50080(10)
b/Å	7.54240(10)
c/Å	18.8223(2)
α/°	90
β/°	107.3970(10)
γ/°	90
Volume/Å^3	2099.91(4)
Z	2
ρ_{calc} g/cm^3	1.390
μ/mm^{-1}	0.889
F(000)	932.0
Crystal size/mm^3	0.09 × 0.06 × 0.05
Radiation	CuKα (λ = 1.54184)
2Θ range for data collection/°	8.808 to 148.594
Index ranges	$-18 \leq h \leq 19$, $-9 \leq k \leq 9$, $-23 \leq l \leq 21$
Reflections collected	25362
Independent reflections	8231 [R_{int} = 0.0275, R_{sigma} = 0.0269]
Data/restraints/parameters	8231/2/591
Goodness-of-fit on F^2	0.876
Final R indexes [I>=2σ (I)]	R_1 = 0.0472, wR_2 = 0.1739
Final R indexes [all data]	R_1 = 0.0483, wR_2 = 0.1779
Largest diff. peak/hole / e Å^{-3}	1.34/-0.64
Flack parameter	0.04(4)

1.2 Computational data

Table S3. Energy analysis for the Conformers of **3**.

Compound	Conformation	E (Hartree)	E (Kcal/mol)	ΔE (Kcal/mol)	Boltzmann Dist (%)
**3**	**3-1**	-997.93751717	-626208.686	0	100%

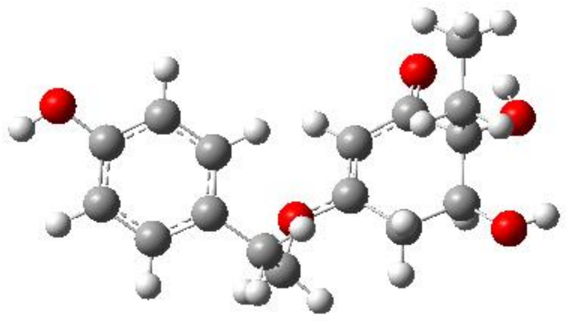

3-1

Figure S1. B3LYP/6-31+G (d,p) optimized low-energy conformers of **3**

Table S4. Energy analysis for the Conformers of **4**.

Compound	Conformation	E (Hartree)	E (Kcal/mol)	ΔE (Kcal/mol)	Boltzmann Dist (%)
**4**	**4-1**	-766.69218509	-481101.5696	0.626620631	13.18%
	**4-2**	-766.69310227	-481102.1451	0.051086894	34.83%
	**4-3**	-766.69318369	-481102.1962	0	37.97%
	**4-4**	-766.69224439	-481101.6068	0.589411591	14.03%

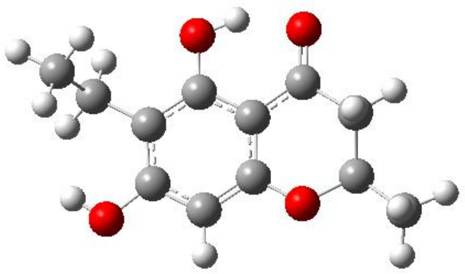

4-1

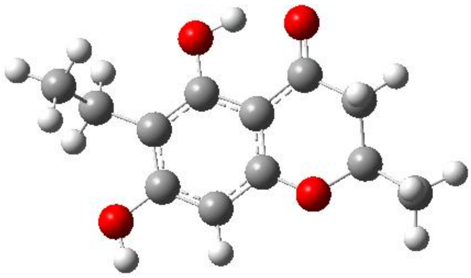

4-2

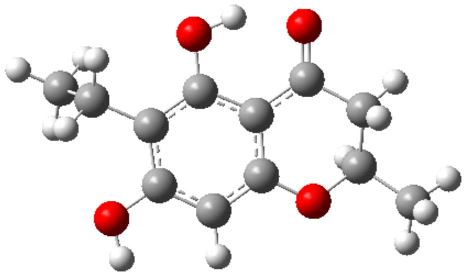

4-3

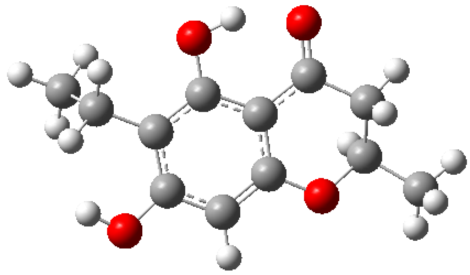

4-4

Figure S2. B3LYP/6-31+G (d,p) optimized low-energy conformers of **4**

2. NMR, HRESIMS, CD, UV and IR spectra of compounds 1-6

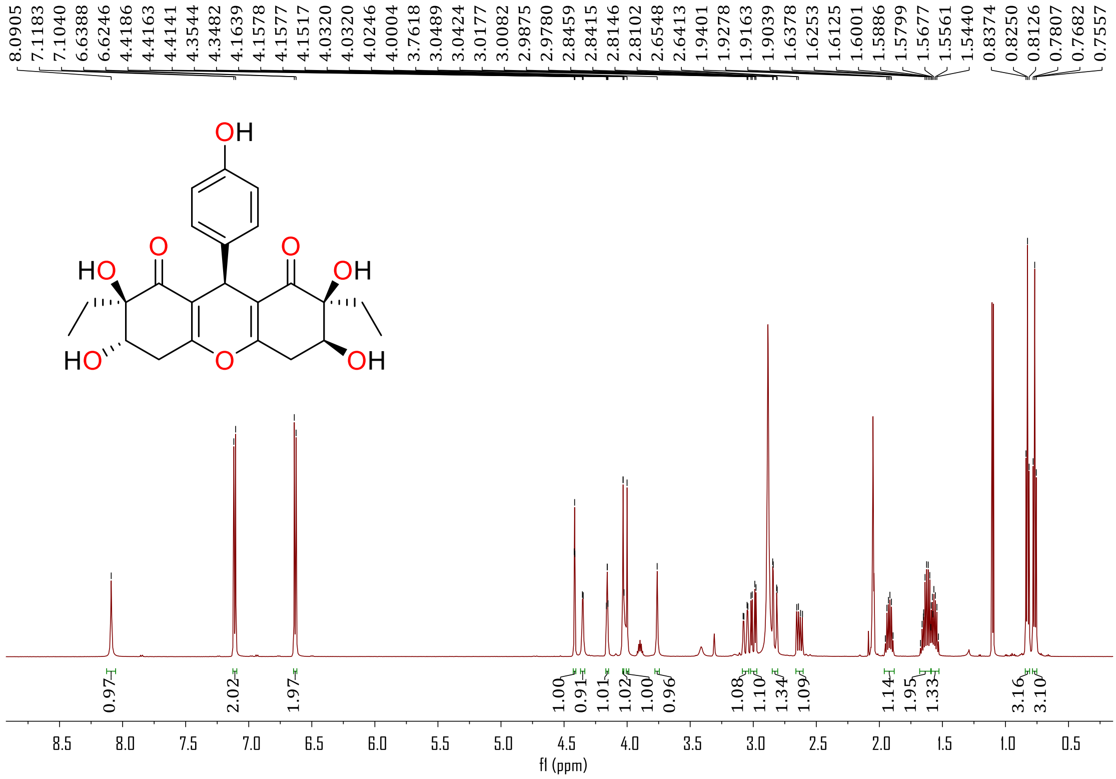

Figure S3. ^{1}H NMR spectrum (600 MHz, CD_3COCD_3) of **1**

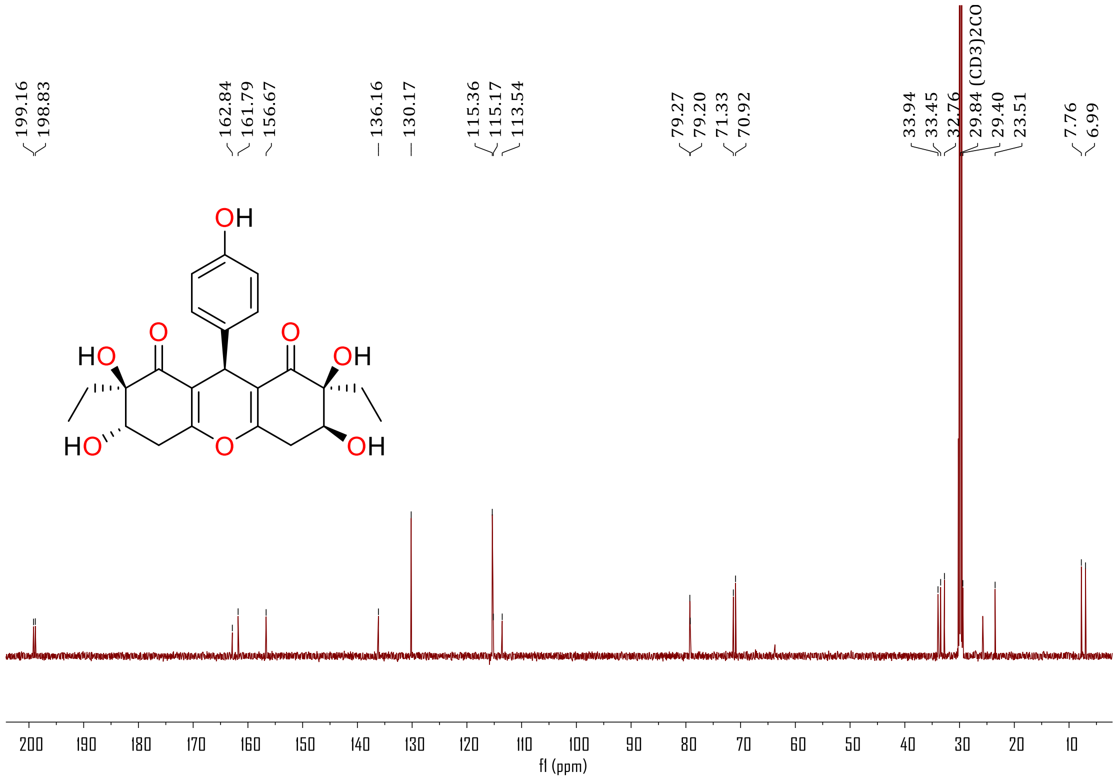

Figure S4. ^{13}C NMR spectrum (150 MHz, CD_3COCD_3) of **1**

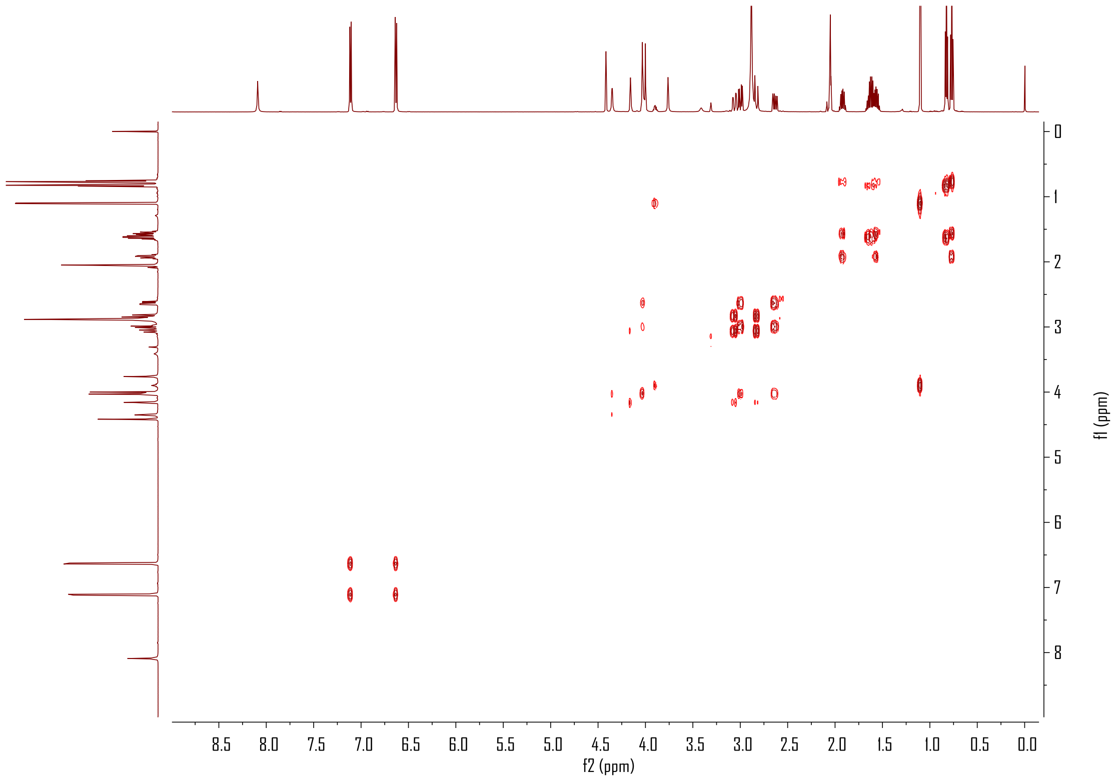

Figure S5. ^{1}H-^{1}H COSY spectrum of **1** in CD_3COCD_3

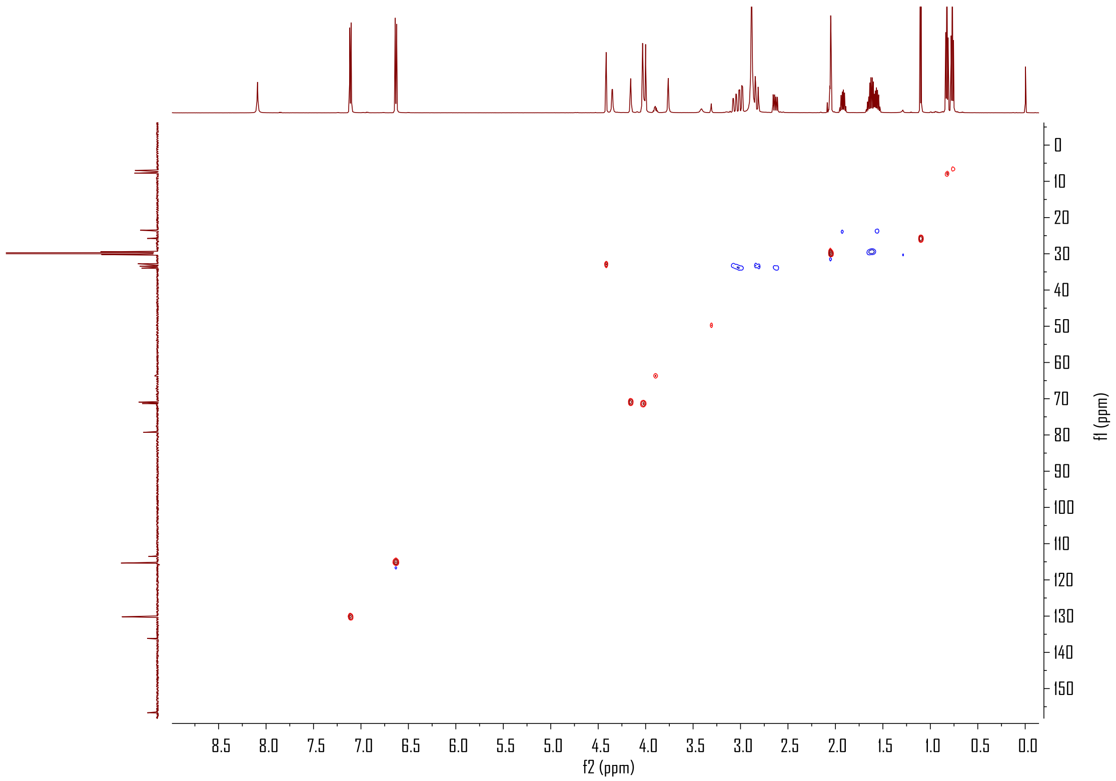

Figure S6. HSQC spectrum of **1** in CD_3COCD_3

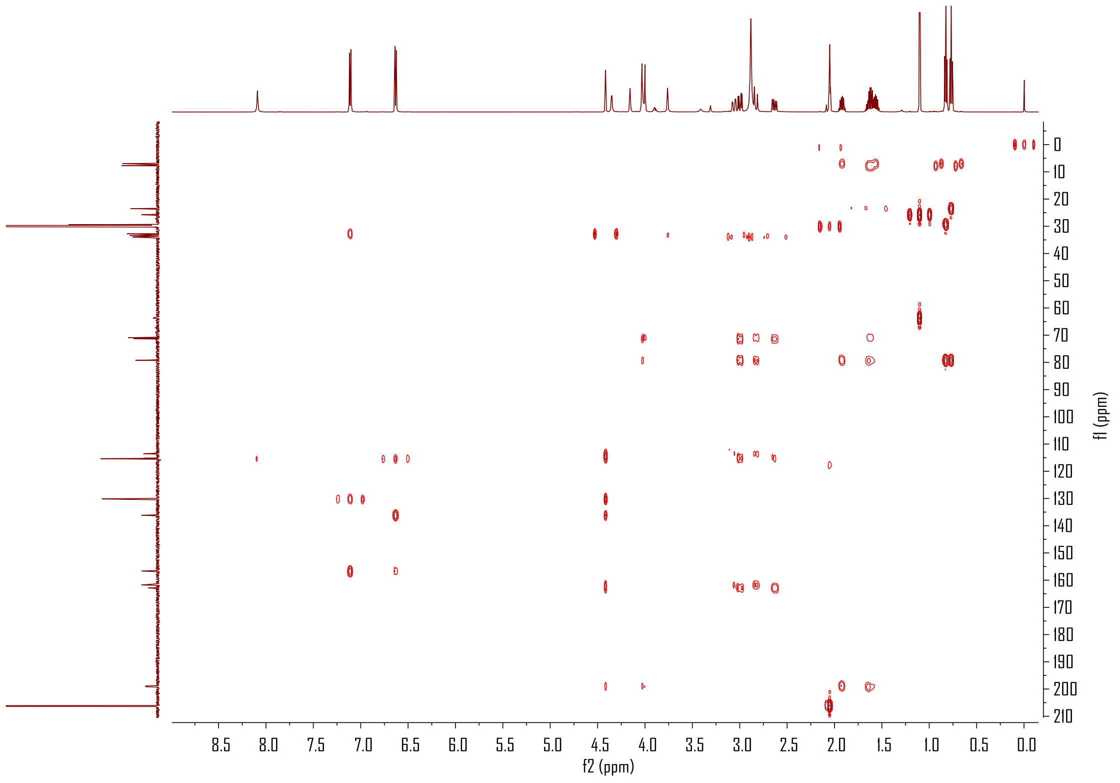

Figure S7. HMBC spectrum of **1** in CD_3COCD_3

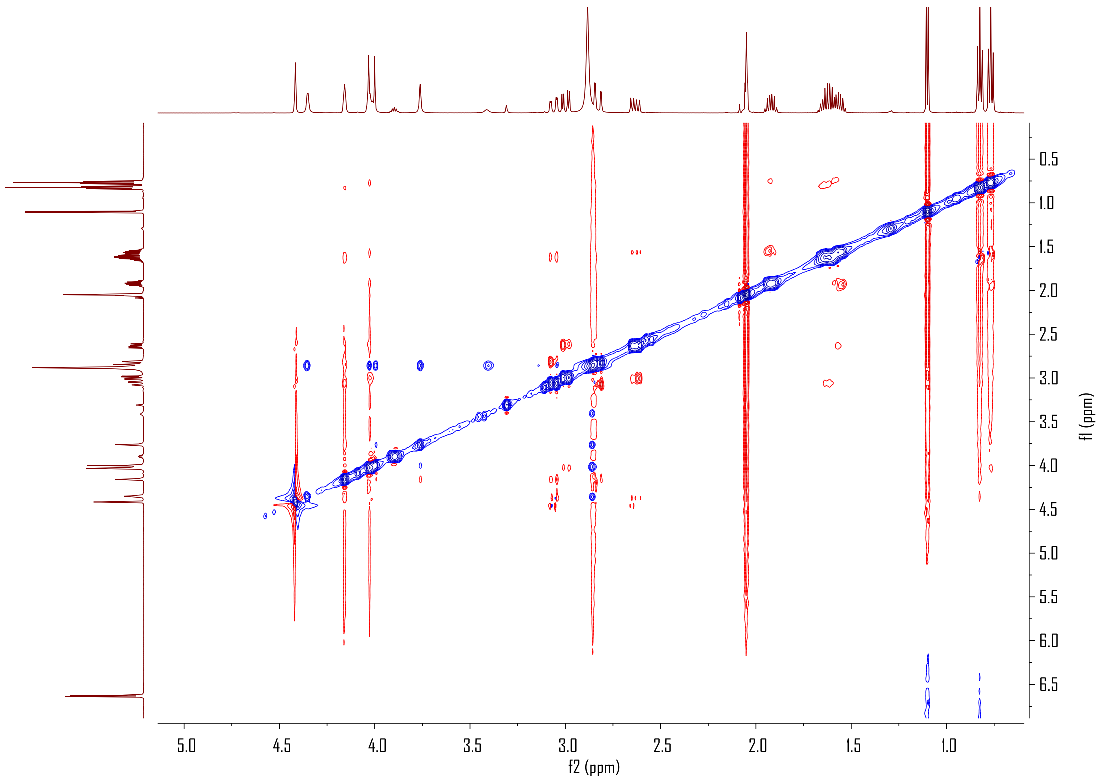

Figure S8. NOESY spectrum of **1** in CD_3COCD_3

Mass Spectrum SmartFormula Report

Analysis Info

Analysis Name	D:\Data\MS\data\201909\chenshanchong_X8-1_pos_3_01_7299.d	Acquisition Date	9/18/2019 4:12:00 PM
Method	LC_Direct Infusion_pos_70-500mz.m	Operator	SCSIO
Sample Name	chenshanchong_X8-1_pos	Instrument	maXis 255552.00029
Comment			

Acquisition Parameter

Source Type	ESI	Ion Polarity	Positive	Set Nebulizer	0.4 Bar
Focus	Active	Set Capillary	4500 V	Set Dry Heater	180 °C
Scan Begin	70 m/z	Set End Plate Offset	-500 V	Set Dry Gas	4.0 l/min
Scan End	1500 m/z	Set Charging Voltage	0 V	Set Divert Valve	Waste
		Set Corona	0 nA	Set APCI Heater	0 °C

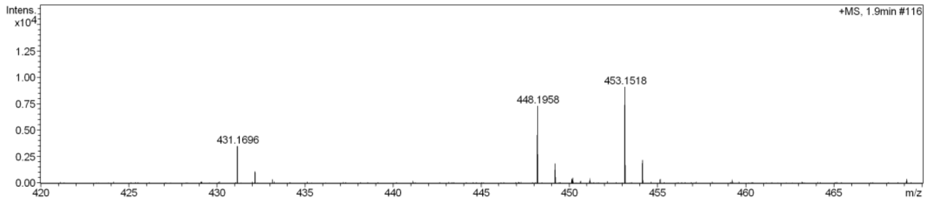

Meas. m/z	#	Ion Formula	Score	m/z	err [ppm]	err [mDa]	mSigma	rdb	e⁻ Conf	N-Rule
431.1696	1	C23H27O8	100.00	431.1700	-1.1	-0.5	39.9	10.5	even	ok
453.1518	1	C23H26NaO8	100.00	453.1520	0.4	0.2	8.2	10.5	even	ok
861.3326	1	C46H53O16	100.00	861.3328	-0.2	-0.2	15.6	20.5	even	ok
883.3149	1	C46H52NaO16	100.00	883.3148	-0.1	-0.1	16.0	20.5	even	ok

chenshanchong_X8-1_pos_3_01_7299.d

Bruker Compass DataAnalysis 4.1 printed: 9/18/2019 4:16:16 PM by: SCSIO Page 1 of 1

Figure S9. HRESIMS spectrum of **1**

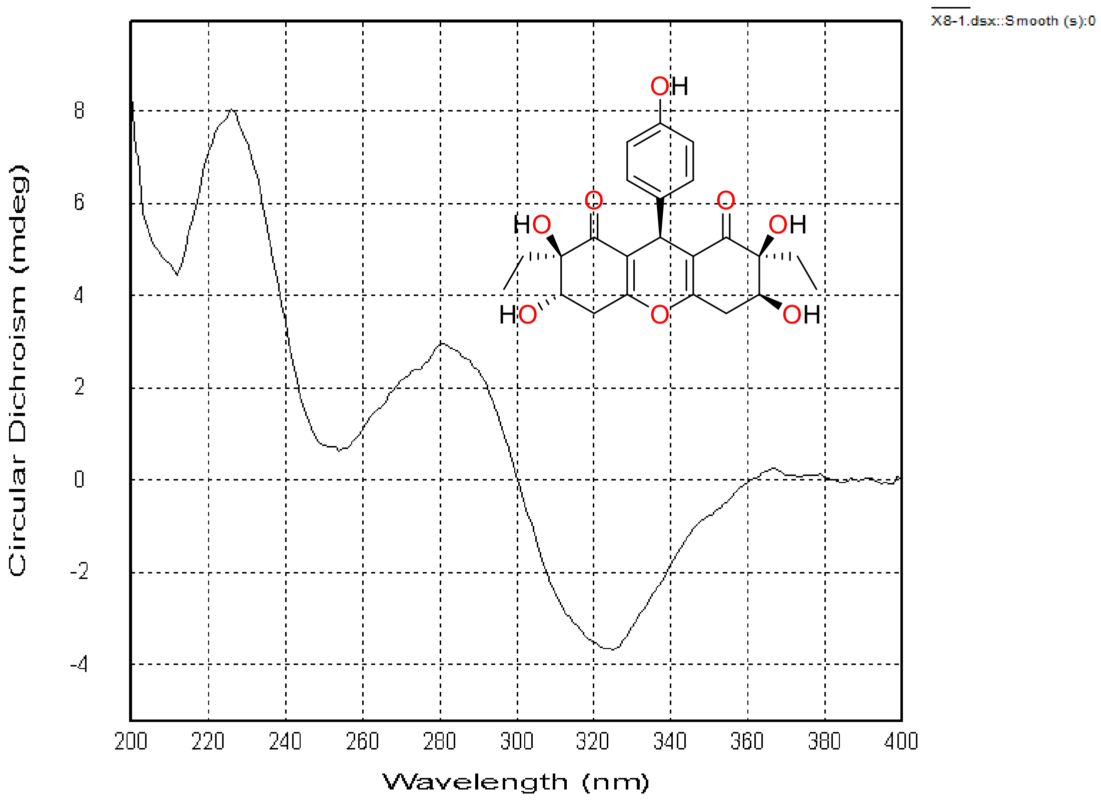

Figure S10. CD spectrum of **1**

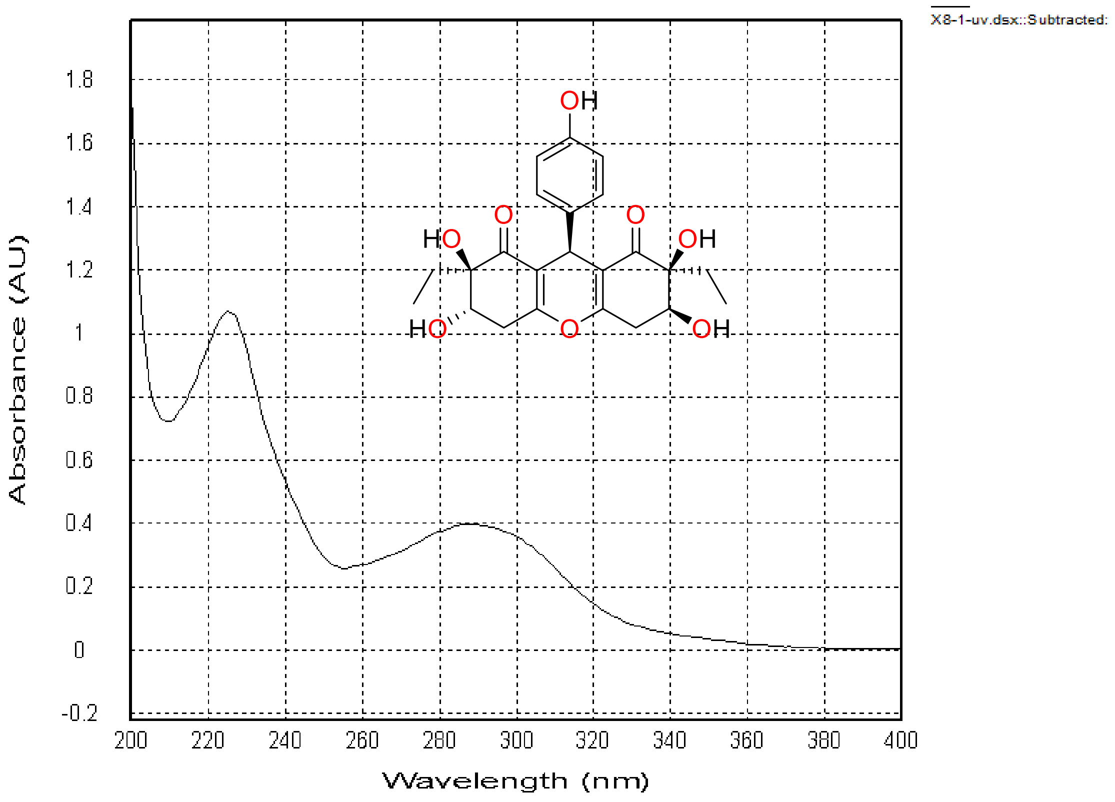

Figure S11. UV spectrum of **1**

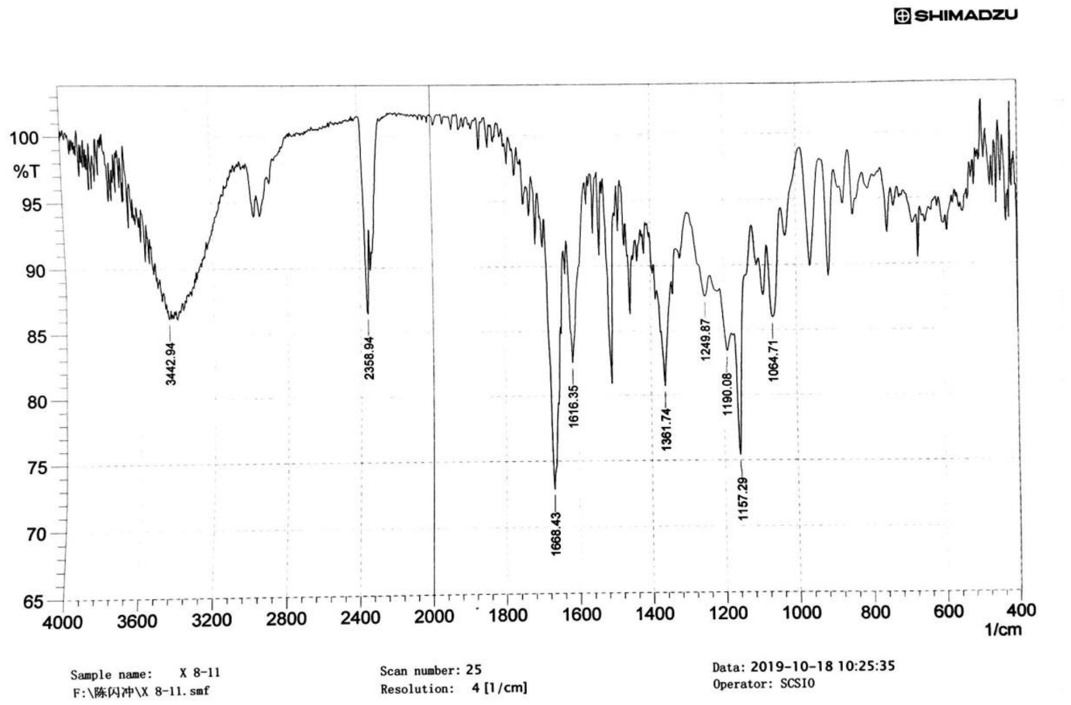

Figure S12. IR spectrum of **1**

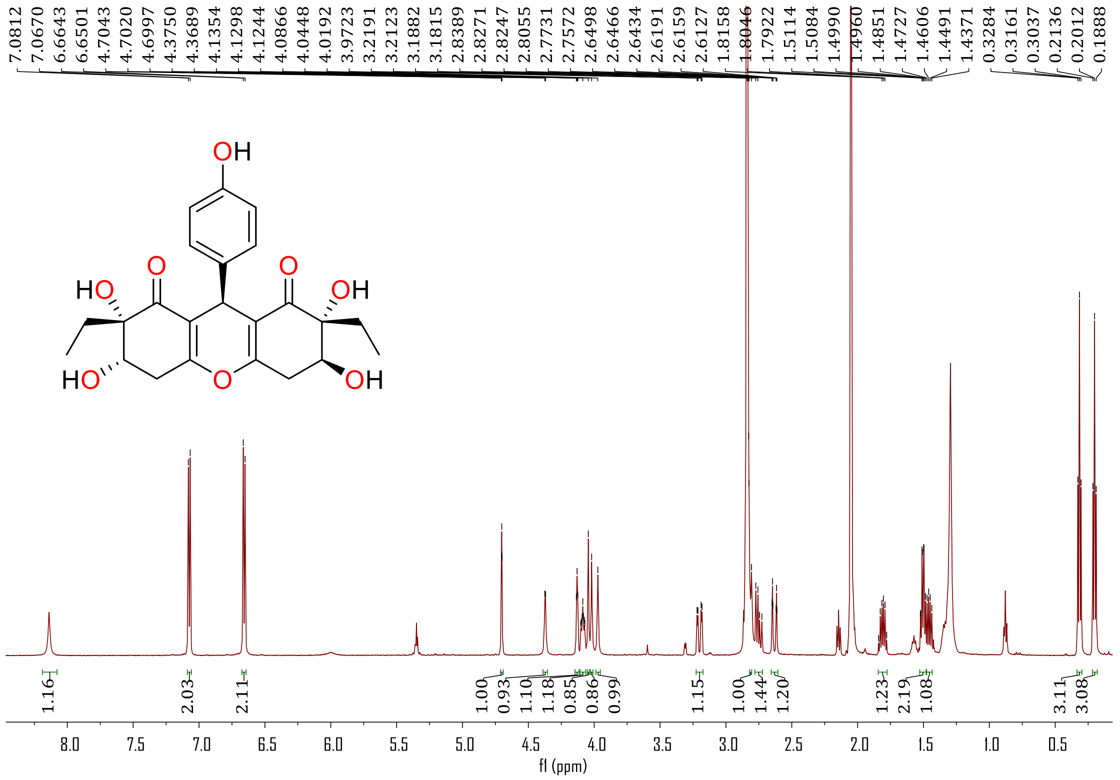

Figure S13. ^{1}H NMR spectrum (600 MHz, CD_3COCD_3) of **2**

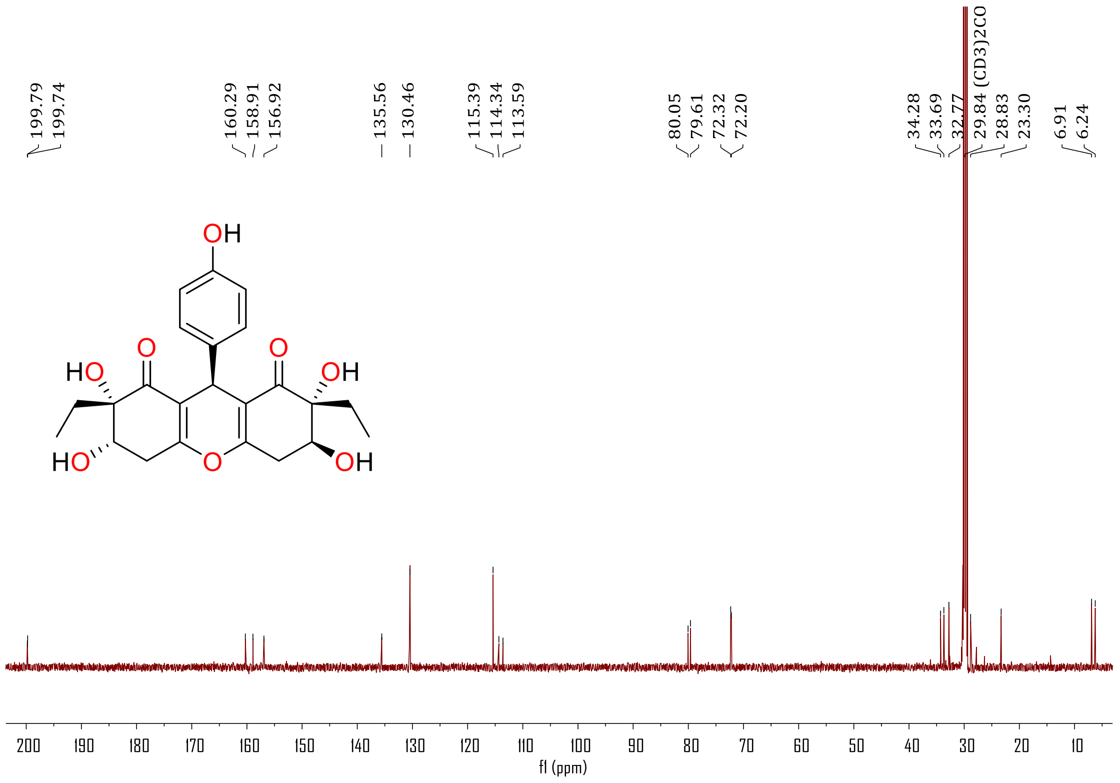

Figure S14. ^{13}C NMR spectrum (150 MHz, CD_3COCD_3) of **2**

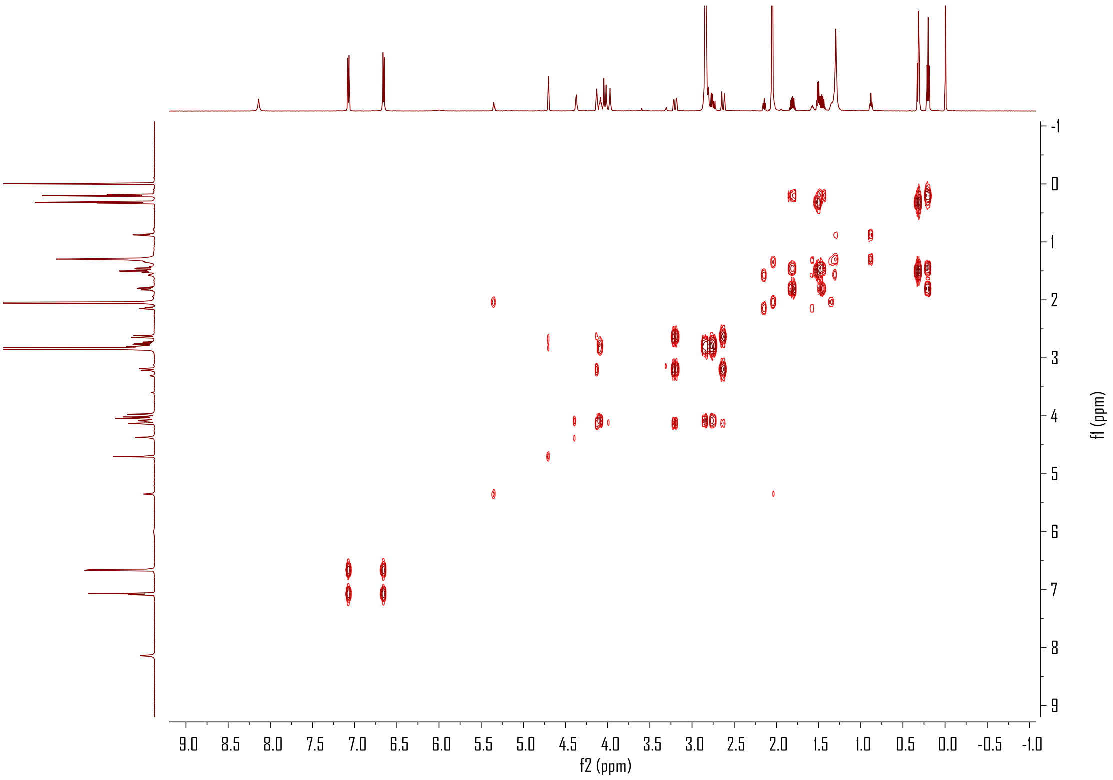

Figure S15. ^{1}H-^{1}H COSY spectrum of **2** in CD_3COCD_3

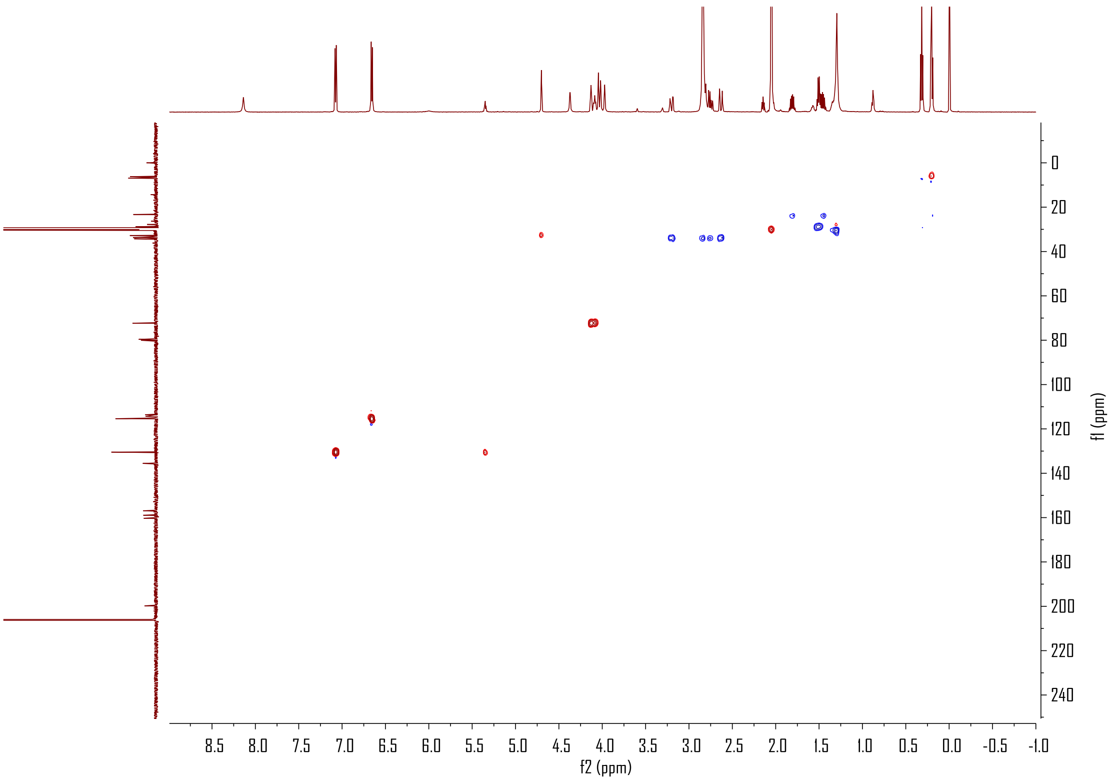

Figure S16. HSQC spectrum of **2** in CD_3COCD_3

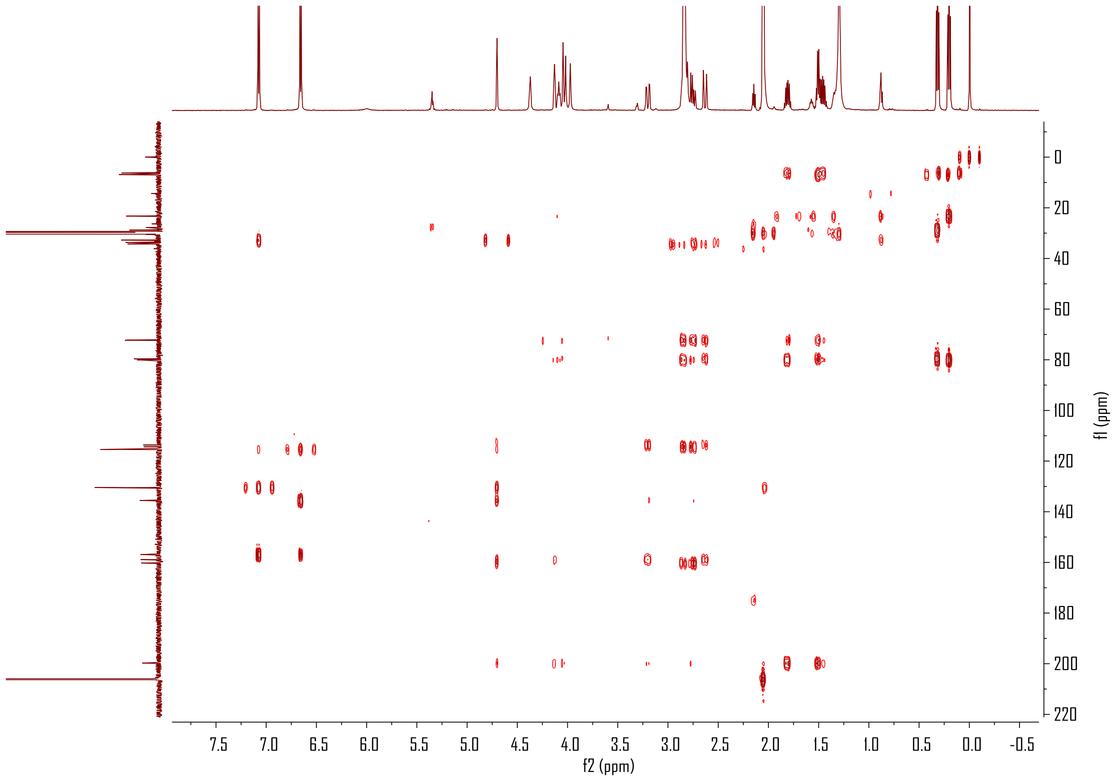

Figure S17. HMBC spectrum of **2** in CD_3COCD_3

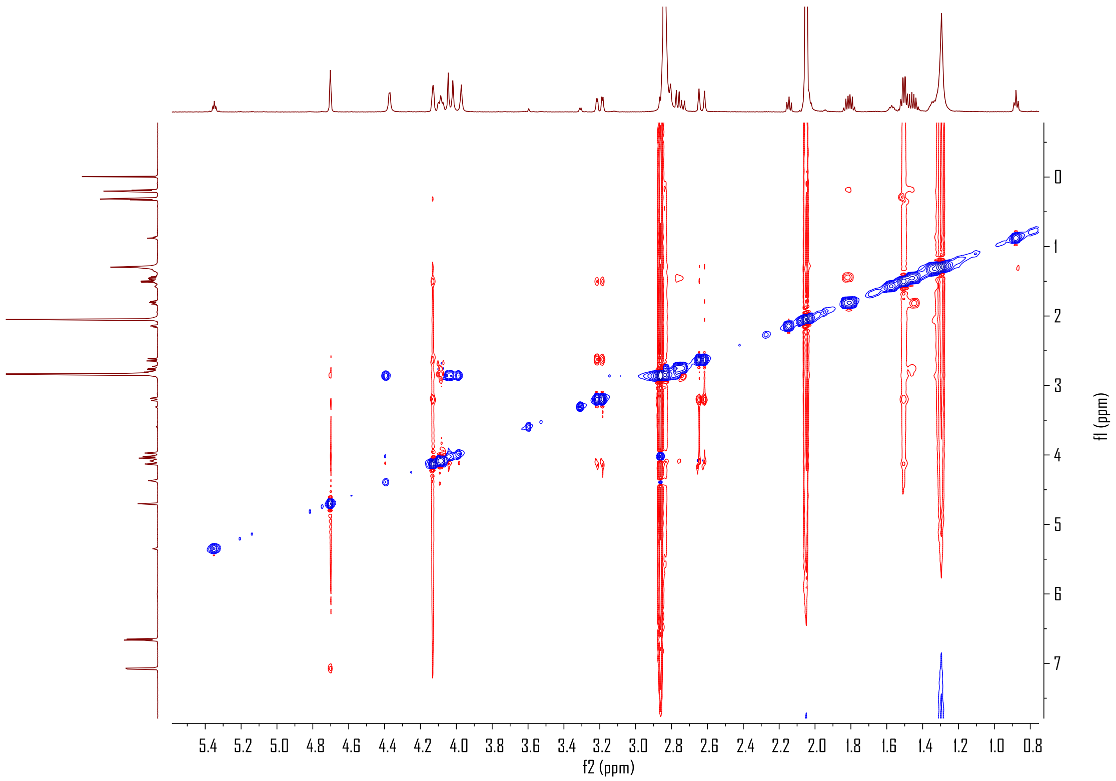

Figure S18. NOESY spectrum of **2** in CD_3COCD_3

Mass Spectrum SmartFormula Report

Analysis Info

Analysis Name	D:\Data\MS\data\201909\chenshanchong_X8-5_neg_14_01_7355.d	Acquisition Date	9/29/2019 9:59:38 AM
Method	LC_Direct Infusion_neg_70-500mz.m	Operator	SCSIO
Sample Name	chenshanchong_X8-5_neg	Instrument	maXis 255552.00029
Comment			

Acquisition Parameter

Source Type	ESI	Ion Polarity	Negative	Set Nebulizer	0.4 Bar
Focus	Active	Set Capillary	4000 V	Set Dry Heater	180 °C
Scan Begin	70 m/z	Set End Plate Offset	-500 V	Set Dry Gas	4.0 l/min
Scan End	1500 m/z	Set Charging Voltage	0 V	Set Divert Valve	Waste
		Set Corona	0 nA	Set APCI Heater	0 °C

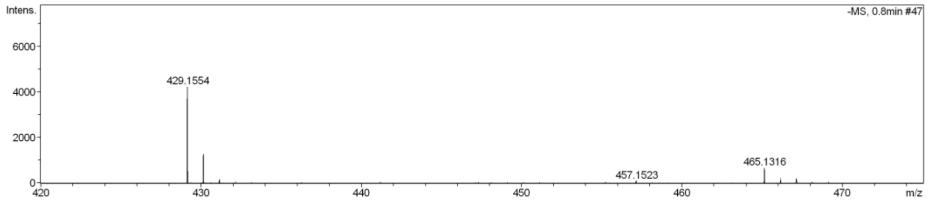

Meas. m/z	#	Ion Formula	Score	m/z	err [ppm]	err [mDa]	mSigma	rdb	e^- Conf	N-Rule
429.1554	1	C23H25O8	100.00	429.1555	0.2	0.1	25.4	11.5	even	ok
465.1316	1	C23H26ClO8	100.00	465.1322	1.2	0.6	35.6	10.5	even	ok
859.3190	1	C46H51O16	100.00	859.3183	-0.9	-0.8	10.4	21.5	even	ok
895.2946	1	C46H52ClO16	100.00	895.2949	-0.4	-0.4	105.4	20.5	even	ok

chenshanchong_X8-5_neg_14_01_7355.d

Bruker Compass DataAnalysis 4.1 printed: 9/29/2019 10:03:43 AM by: SCSIO Page 1 of 1

Figure S19. HRESIMS spectrum of **2**

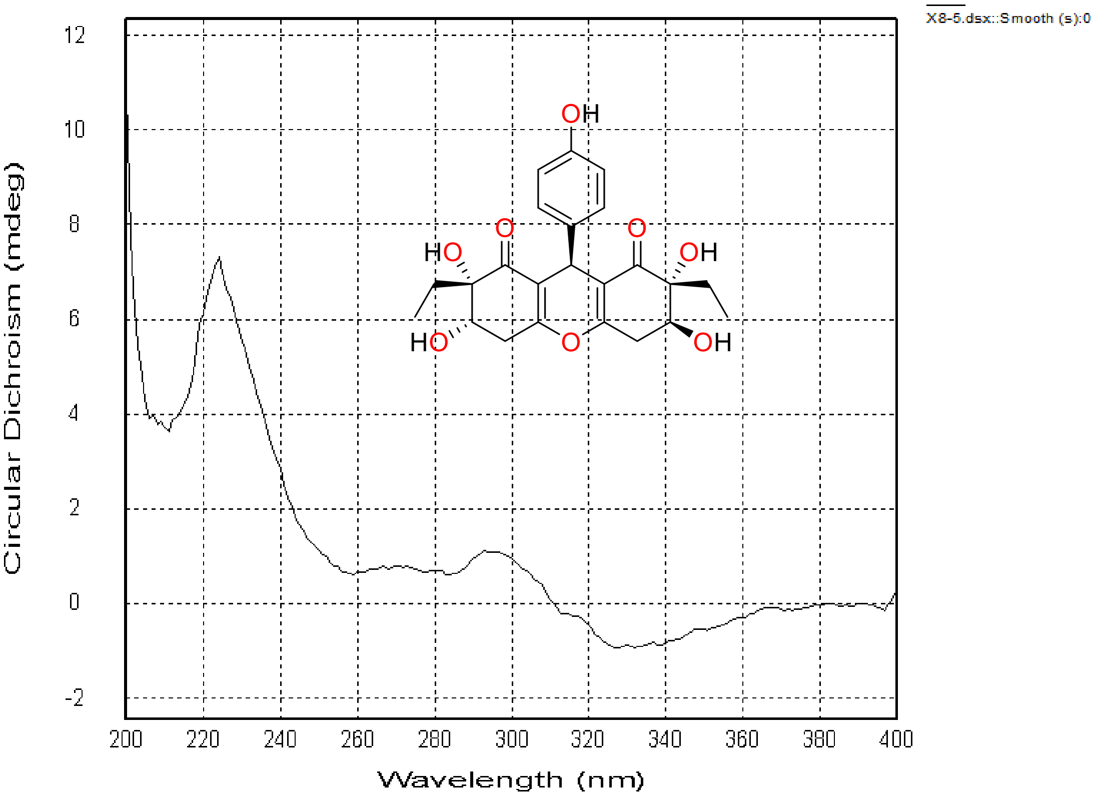

Figure S20. CD spectrum of **2**

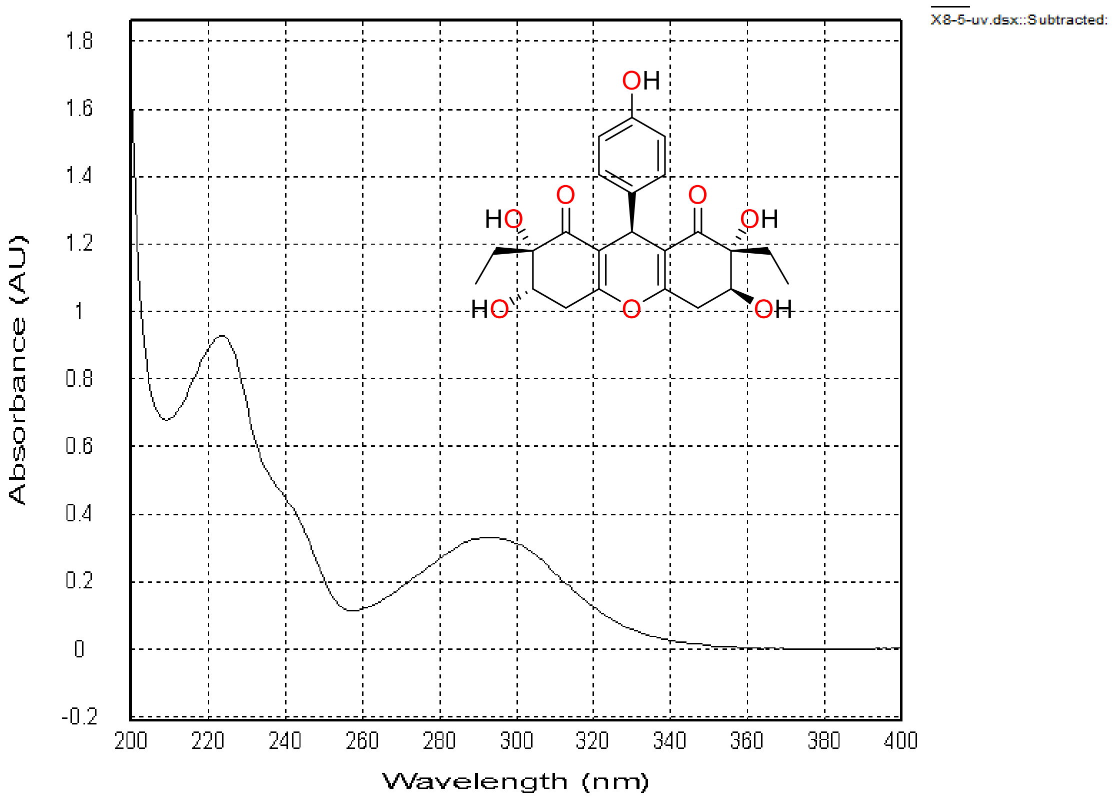

Figure S21. UV spectrum of **2**

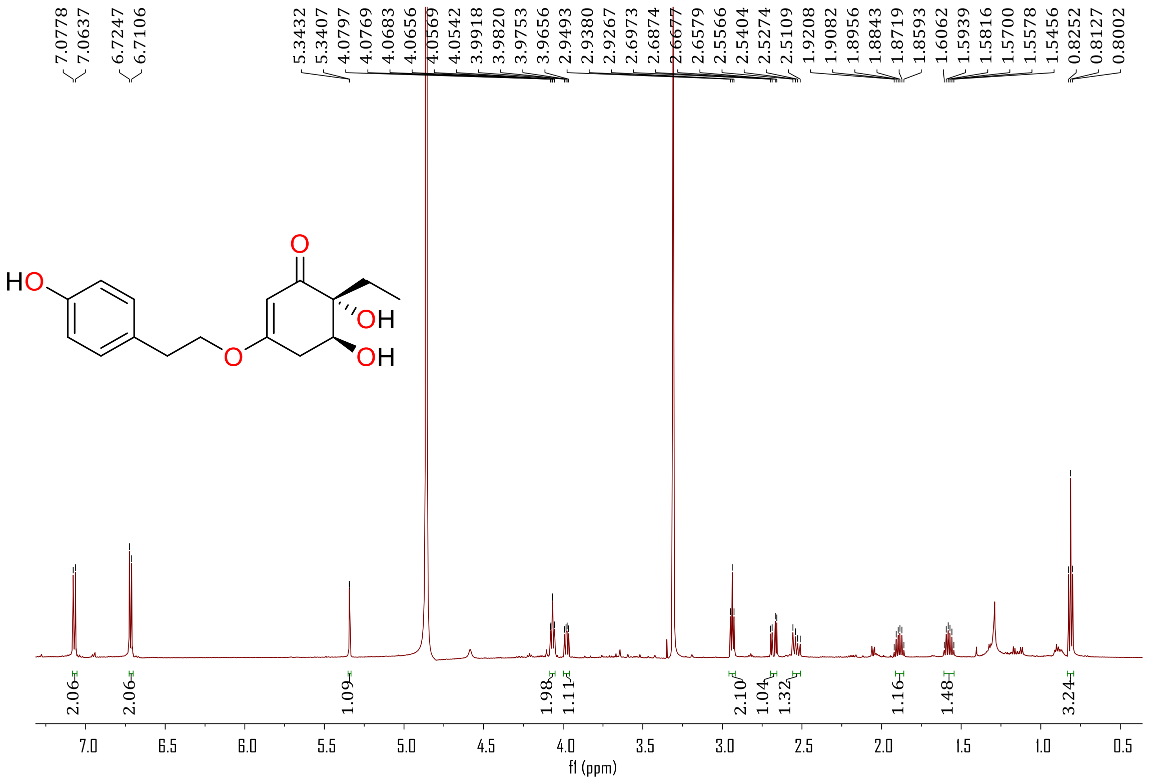

Figure S22. ^{1}H NMR spectrum (600 MHz, CD_3OD) of **3**

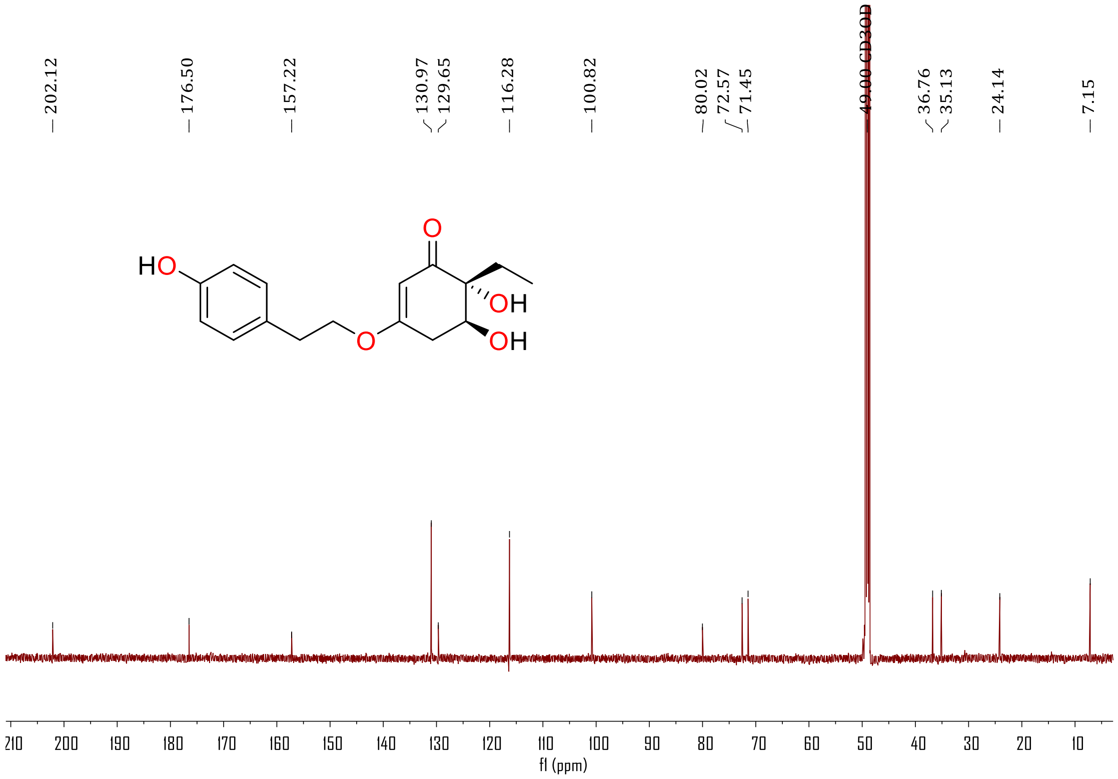

Figure S23. ^{13}C NMR spectrum (150 MHz, CD_3OD) of **3**

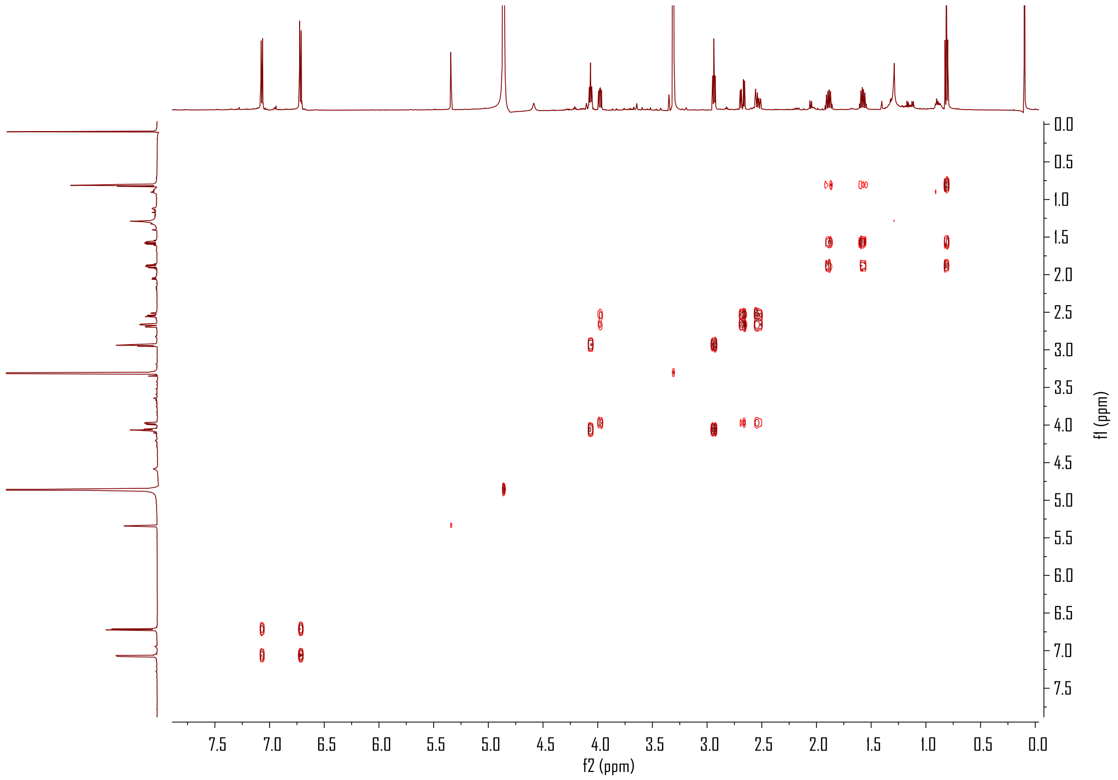

Figure S24. ^{1}H-^{1}H COSY spectrum of **3** in CD_3OD

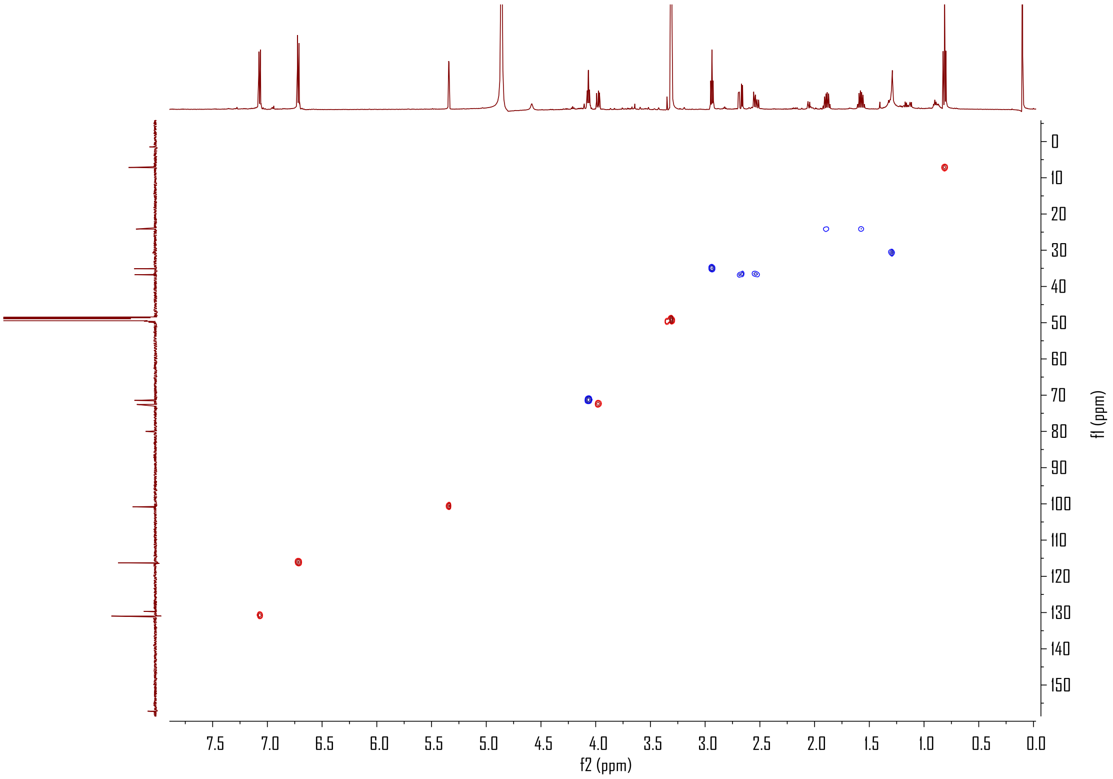

Figure S25. HSQC spectrum of **3** in CD_3OD

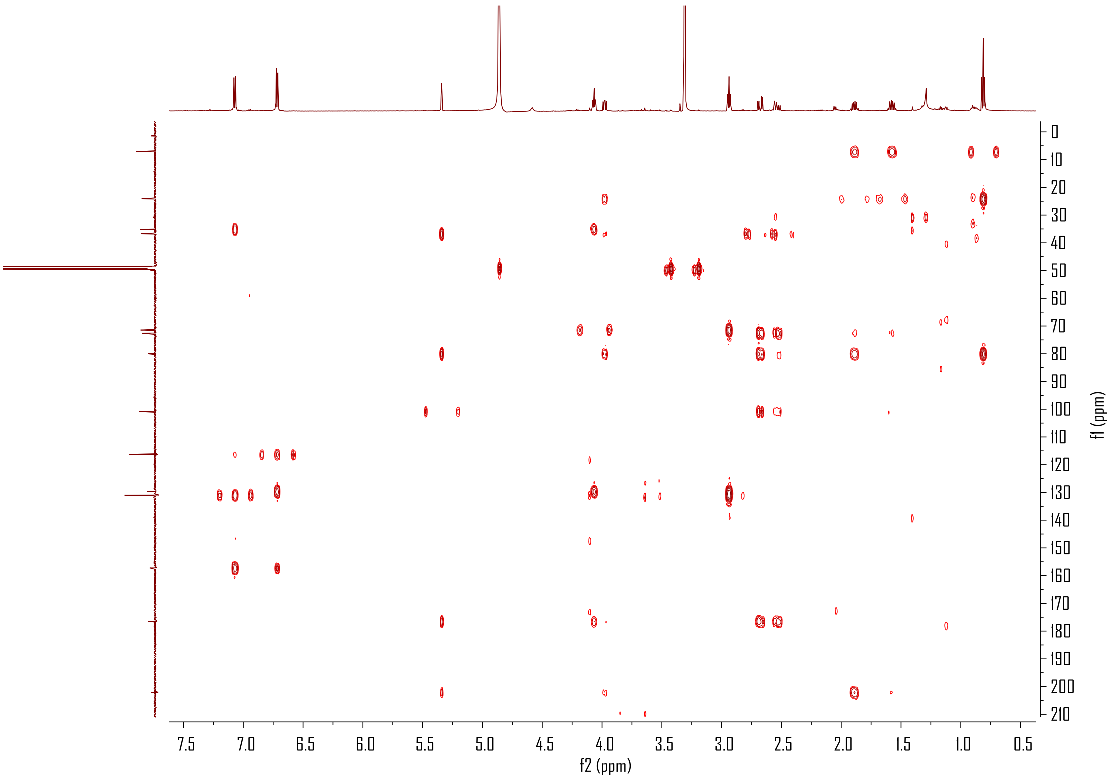

Figure S26. HMBC spectrum of **3** in CD_3OD

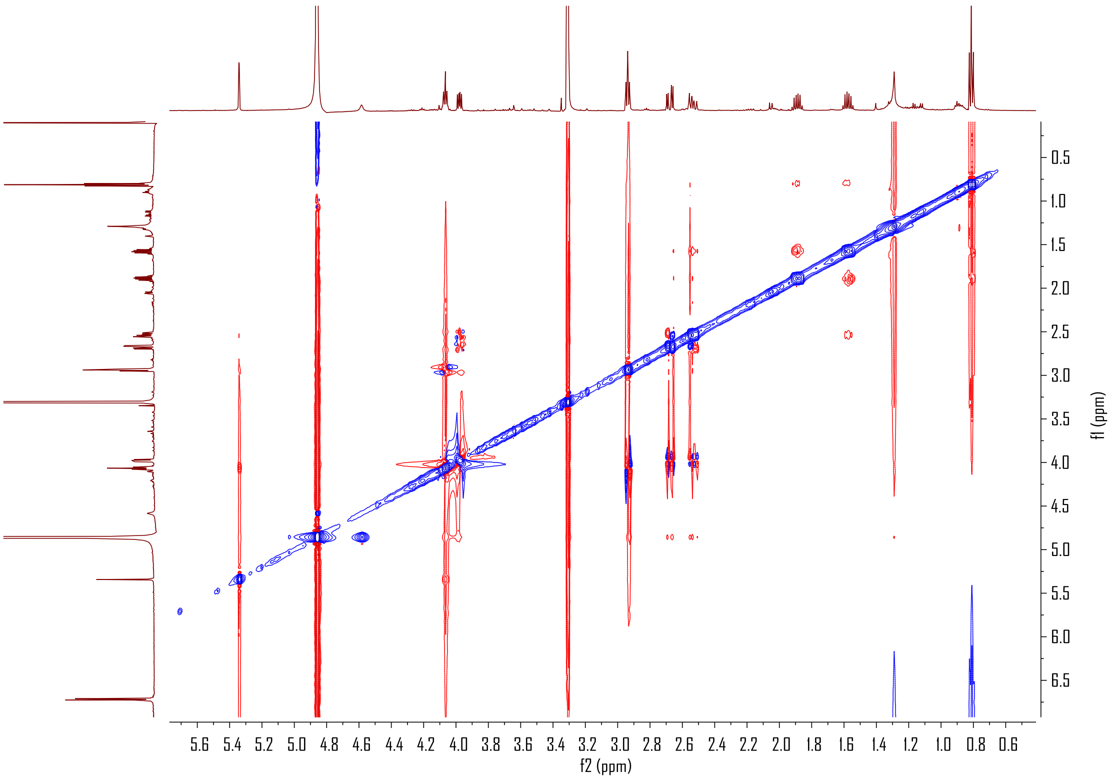

Figure S27. NOESY spectrum of **3** in CD_3OD

Mass Spectrum SmartFormula Report

Analysis Info

Analysis Name D:\Data\MS\data\201908\chenshanchong_X7-12_pos_41_01_7104.d
Method LC_Direct Infusion_pos_70-500mz.m
Sample Name chenshanchong_X7-12_pos
Comment

Acquisition Date 8/19/2019 10:00:16 AM
Operator SCSIO
Instrument maXis 255552.00029

Acquisition Parameter

Source Type	ESI	Ion Polarity	Positive	Set Nebulizer	0.4 Bar
Focus	Active	Set Capillary	4500 V	Set Dry Heater	180 °C
Scan Begin	70 m/z	Set End Plate Offset	-500 V	Set Dry Gas	4.0 l/min
Scan End	1500 m/z	Set Charging Voltage	0 V	Set Divert Valve	Waste
		Set Corona	0 nA	Set APCI Heater	0 °C

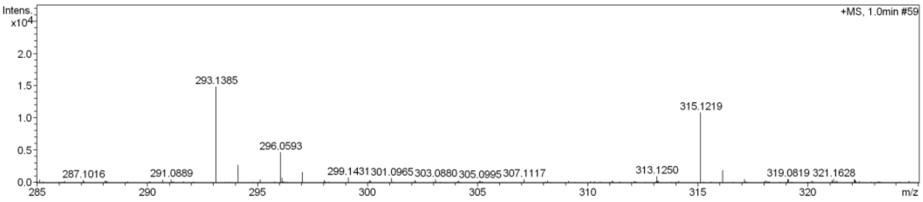

Meas. m/z	#	Ion Formula	Score	m/z	err [ppm]	err [mDa]	mSigma	rdb	e⁻ Conf	N-Rule
293.1385	1	C16H21O5	100.00	293.1384	0.4	0.1	6.1	6.5	even	ok
315.1219	1	C16H20NaO5	64.98	315.1203	-5.2	-1.6	13.4	6.5	even	ok
607.2533	1	C32H40NaO10	100.00	607.2514	-3.2	-1.9	44.1	12.5	even	ok

chenshanchong_X7-12_pos_41_01_7104.d
Bruker Compass DataAnalysis 4.1 printed: 8/19/2019 10:08:48 AM by: SCSIO Page 1 of 1

Figure S28. HRESIMS spectrum of **3**

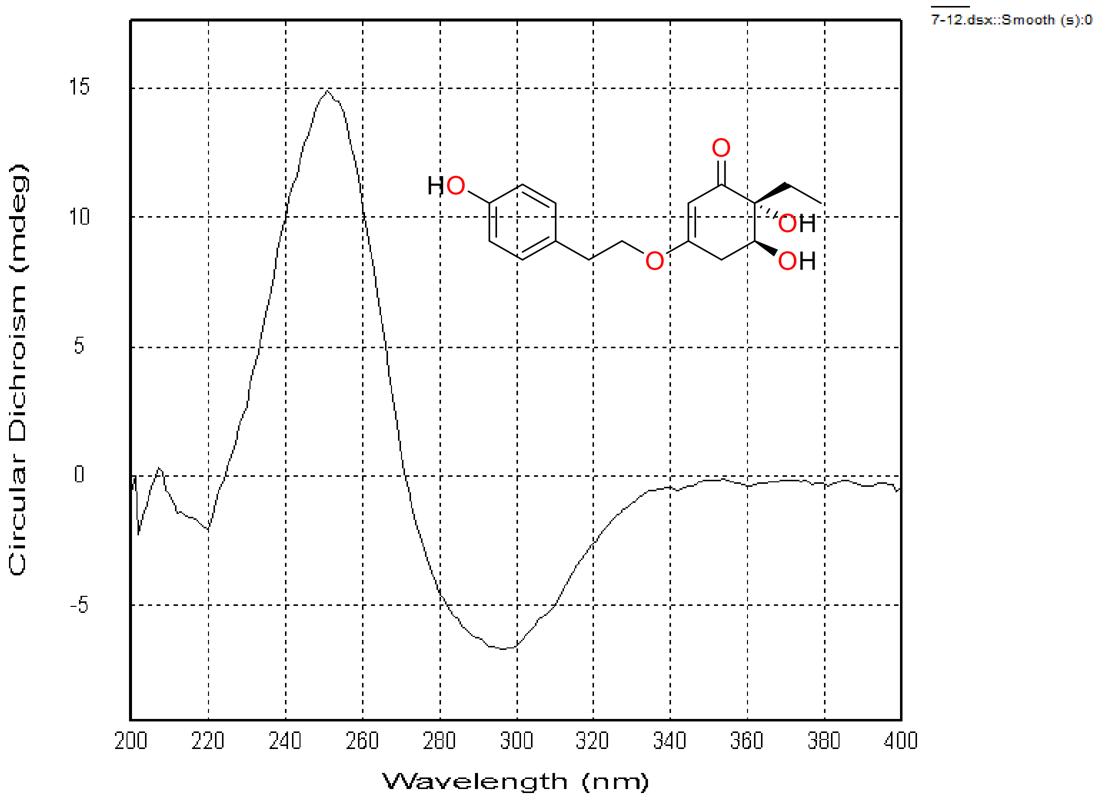

Figure S29. CD spectrum of **3**

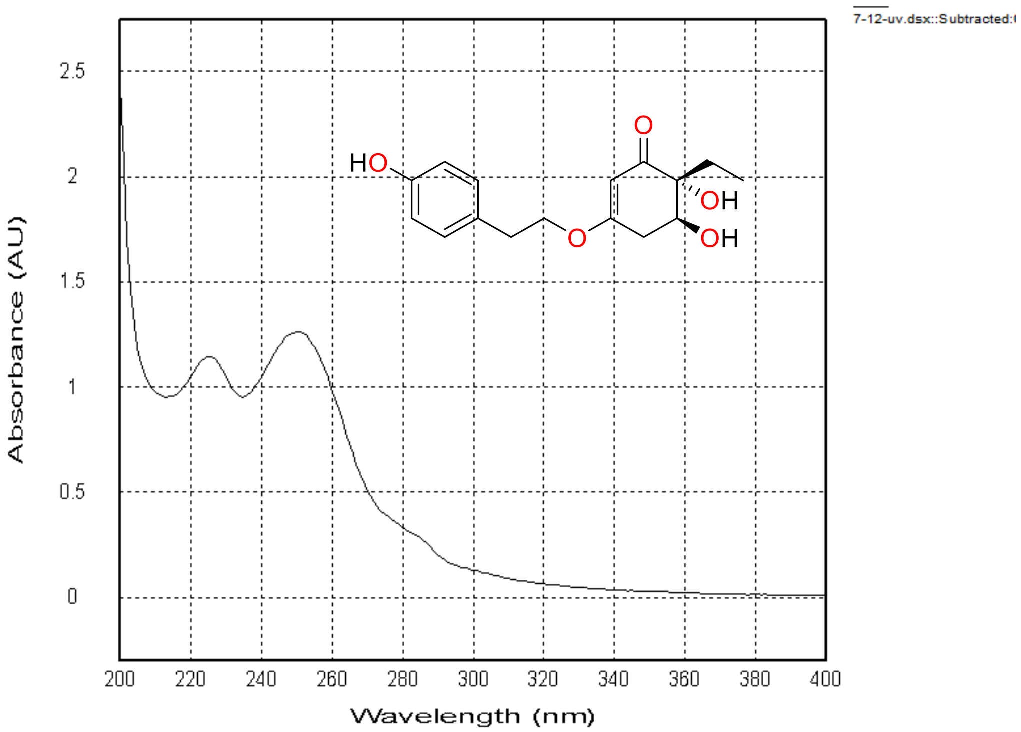

Figure S30. UV spectrum of **3**

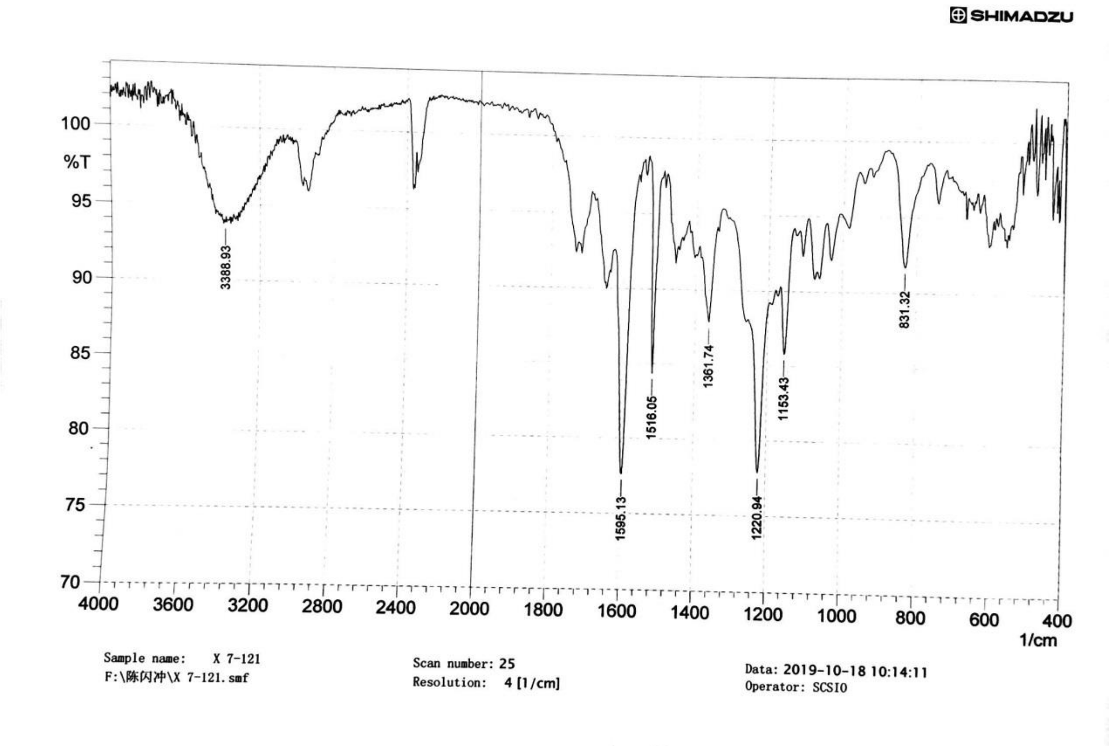

Figure S31. IR spectrum of **3**

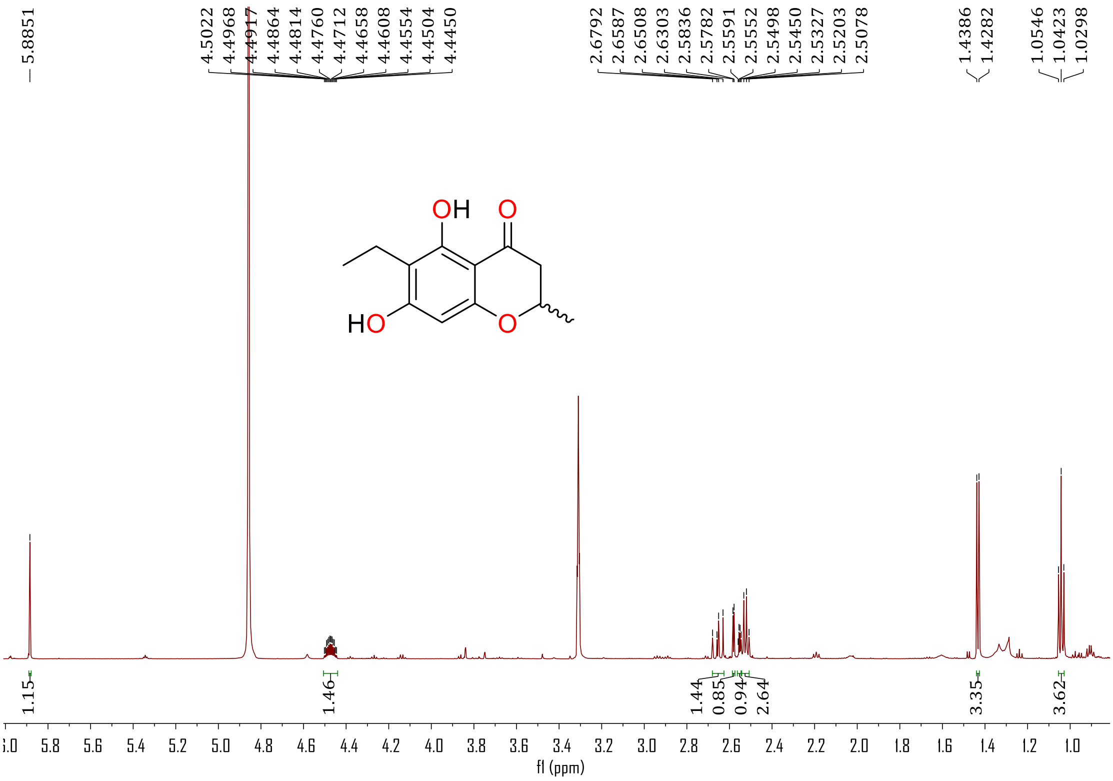

Figure S32. ^{1}H NMR spectrum (600 MHz, CD_3OD) of **4**

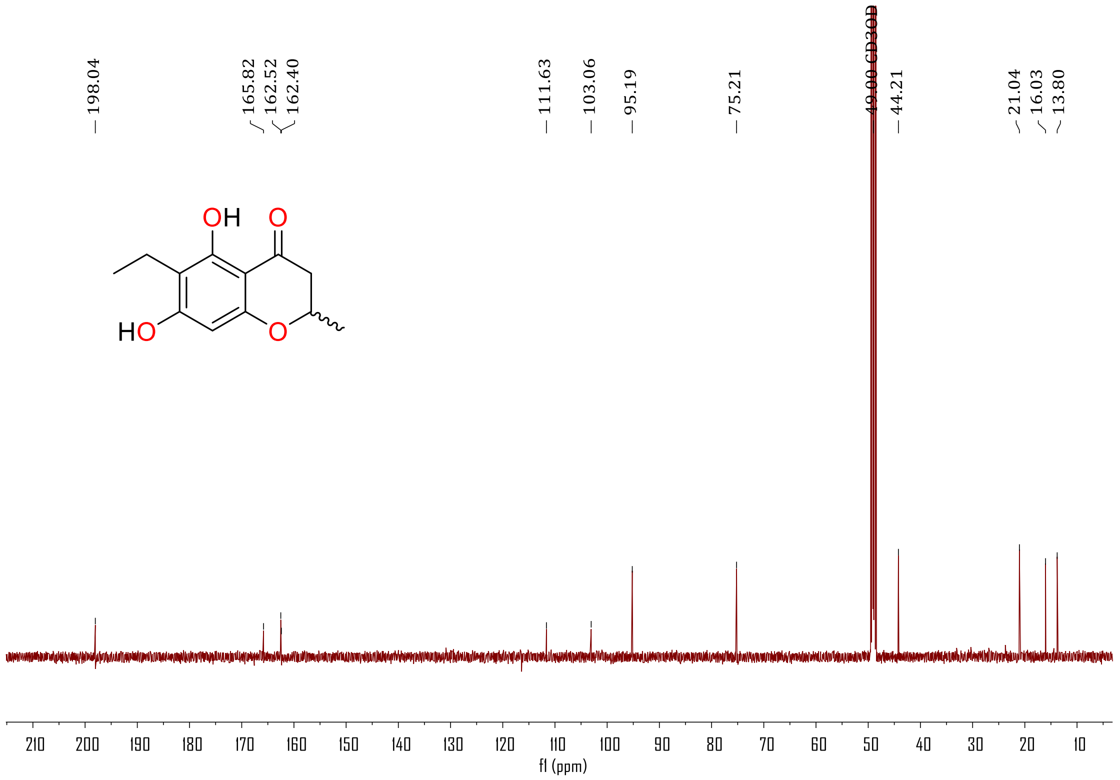

Figure S33. ^{13}C NMR spectrum (150 MHz, CD_3OD) of **4**

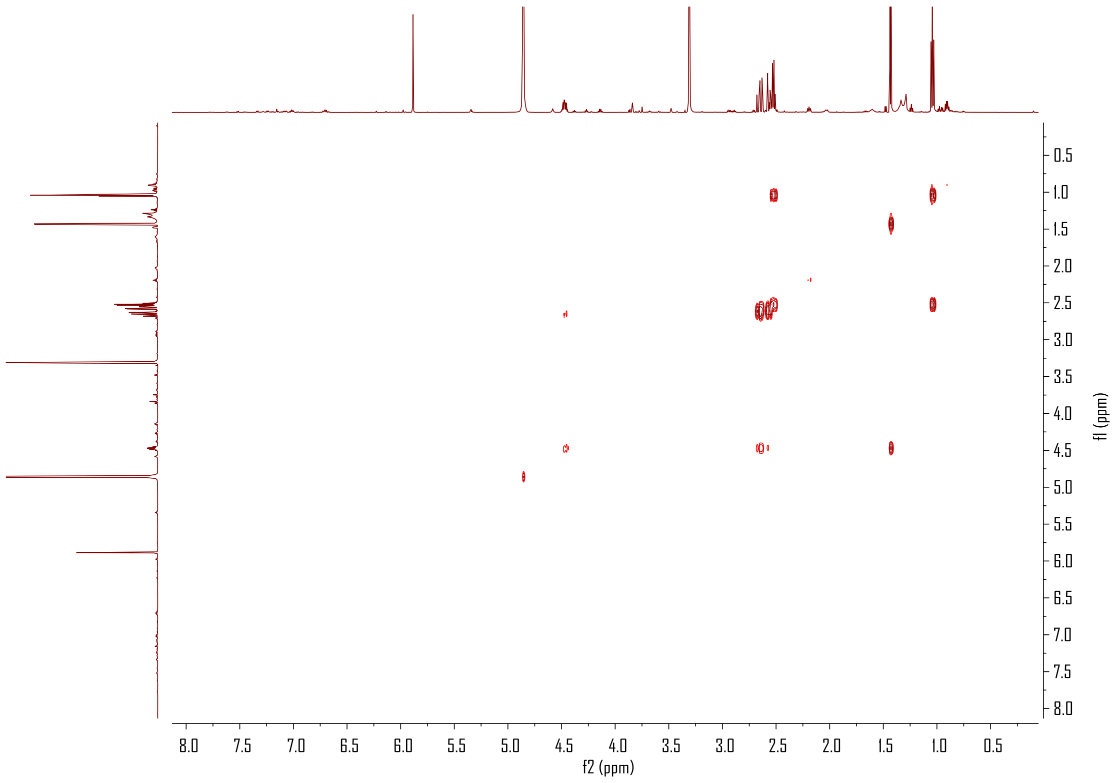

Figure S34. ^{1}H-^{1}H COSY spectrum of **4** in CD_3OD

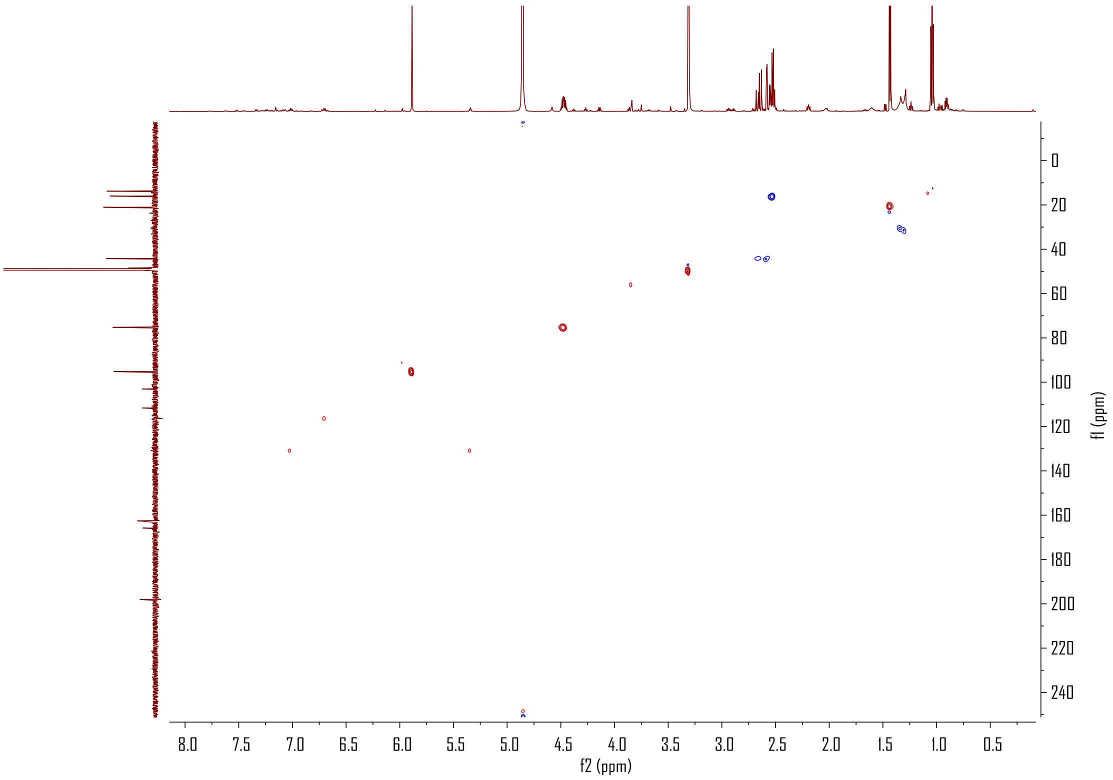

Figure S35. HSQC spectrum of **4** in CD_3OD

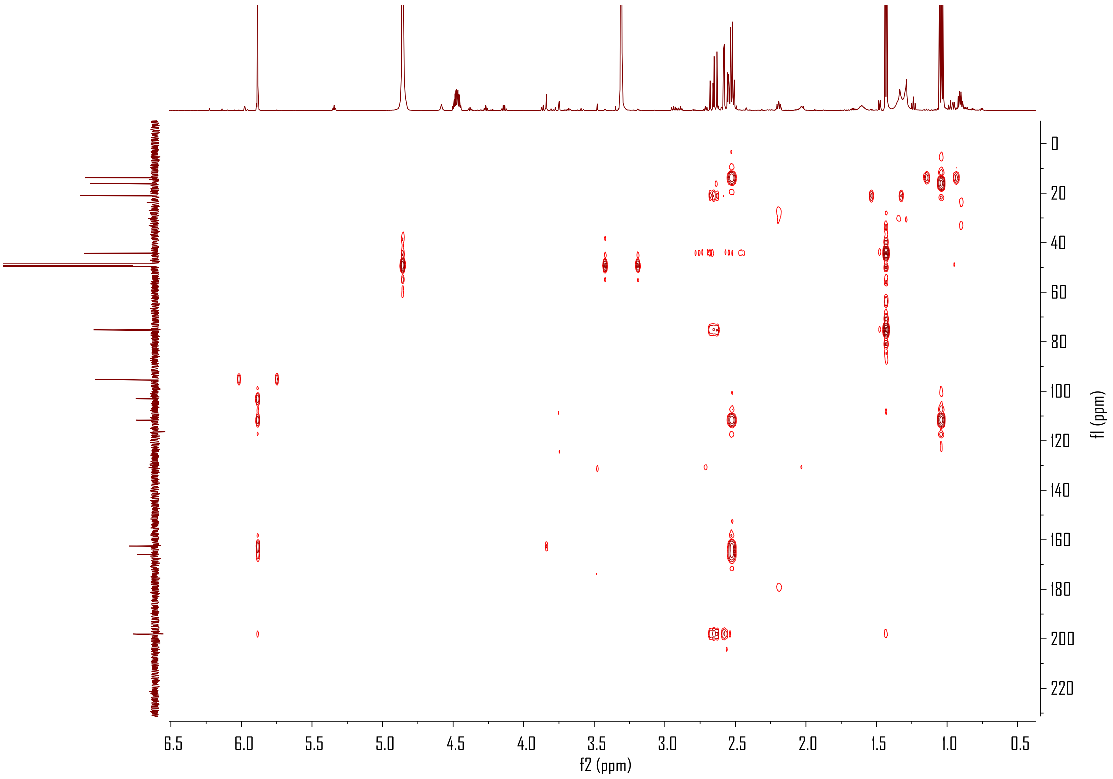

Figure S36. HMBC spectrum of **4** in CD_3OD

Mass Spectrum SmartFormula Report

Analysis Info

Analysis Name	D:\Data\MS\data\201908\chenshanchong_X 5-1_pos_41_01_7076.d	Acquisition Date	8/13/2019 3:46:21 PM	
Method	LC_Direct Infusion_pos_70-500mz.m	Operator	SCSIO	
Sample Name	chenshanchong_X 5-1_pos	Instrument	maXis	255552.00029
Comment				

Acquisition Parameter

Source Type	ESI	Ion Polarity	Positive	Set Nebulizer	0.4 Bar
Focus	Active	Set Capillary	4500 V	Set Dry Heater	180 °C
Scan Begin	70 m/z	Set End Plate Offset	-500 V	Set Dry Gas	4.0 l/min
Scan End	1500 m/z	Set Charging Voltage	0 V	Set Divert Valve	Waste
		Set Corona	0 nA	Set APCI Heater	0 °C

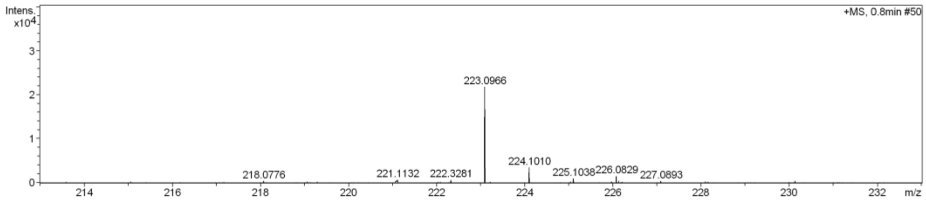

Meas. m/z	#	Ion Formula	Score	m/z	err [ppm]	err [mDa]	mSigma	rdb	e⁻ Conf	N-Rule
223.0966	1	C12H15O4	100.00	223.0965	-0.7	-0.1	23.0	5.5	even	ok

chenshanchong_X 5-1_pos_41_01_7076.d

Bruker Compass DataAnalysis 4.1 printed: 8/13/2019 3:54:04 PM by: SCSIO Page 1 of 1

Figure S37. HRESIMS spectrum of **4a**

Mass Spectrum SmartFormula Report

Analysis Info

Analysis Name	D:\Data\MS\data\201909\chenshanchong_X5-1-2_neg_11_01_7356.d	Acquisition Date	9/29/2019 10:03:05 AM	
Method	LC_Direct Infusion_neg_70-500mz.m	Operator	SCSIO	
Sample Name	chenshanchong_X5-1-2_neg	Instrument	maXis	255552.00029
Comment				

Acquisition Parameter

Source Type	ESI	Ion Polarity	Negative	Set Nebulizer	0.4 Bar
Focus	Active	Set Capillary	4000 V	Set Dry Heater	180 °C
Scan Begin	70 m/z	Set End Plate Offset	-500 V	Set Dry Gas	4.0 l/min
Scan End	1500 m/z	Set Charging Voltage	0 V	Set Divert Valve	Waste
		Set Corona	0 nA	Set APCI Heater	0 °C

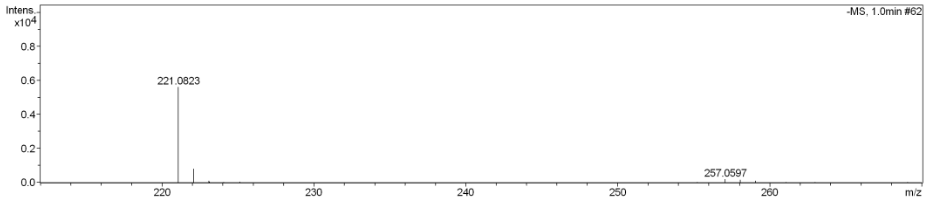

Meas. m/z	#	Ion Formula	Score	m/z	err [ppm]	err [mDa]	mSigma	rdb	e⁻ Conf	N-Rule
221.0823	1	C12H13O4	100.00	221.0819	-1.6	-0.4	7.1	6.5	even	ok
257.0597	1	C12H14ClO4	100.00	257.0586	-4.1	-1.0	333.7	5.5	even	ok

chenshanchong_X5-1-2_neg_11_01_7356.d

Bruker Compass DataAnalysis 4.1 printed: 9/29/2019 10:07:10 AM by: SCSIO Page 1 of 1

Figure S38. HRESIMS spectrum of **4b**

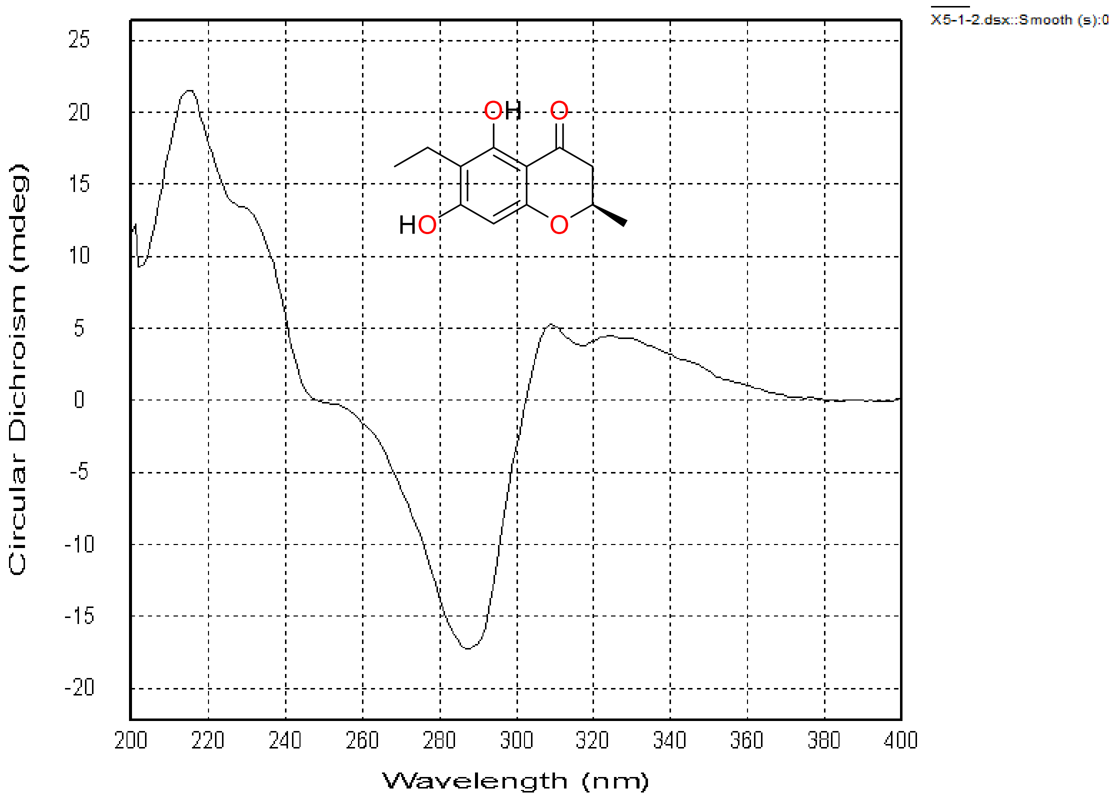

Figure S39. CD spectrum of **4a**

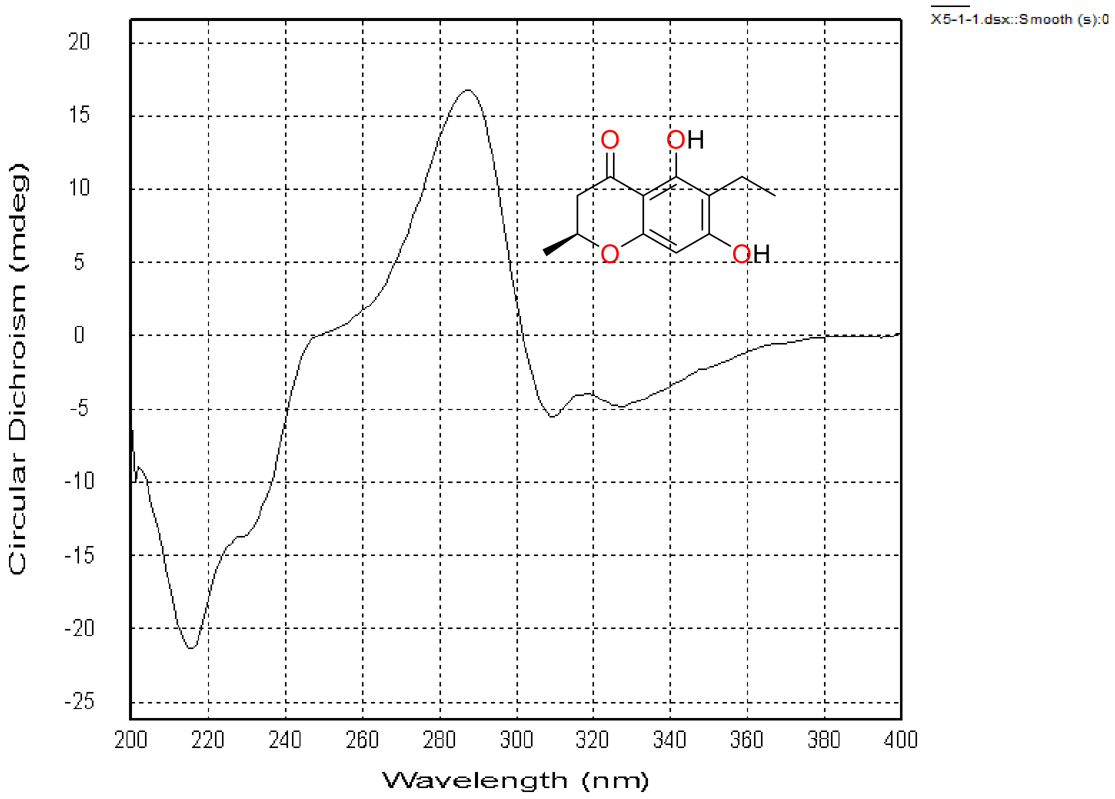

Figure S40. CD spectrum of **4b**

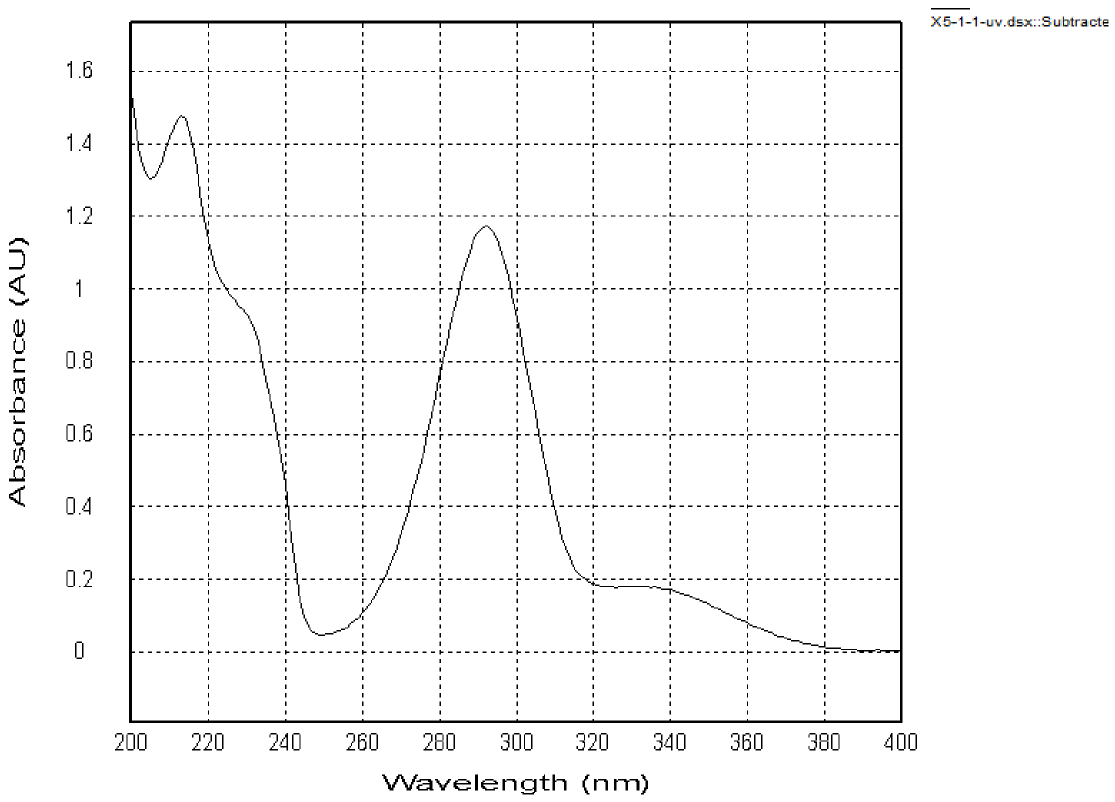

Figure S41. UV spectrum of **4**

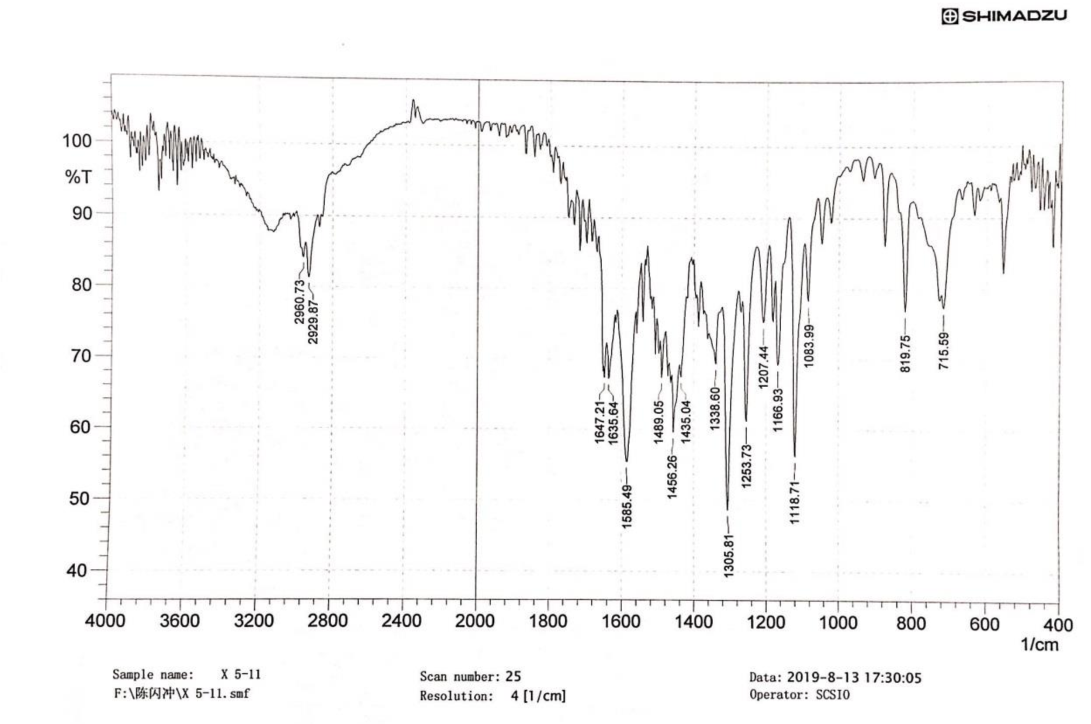

Figure S42. IR spectrum of **4**

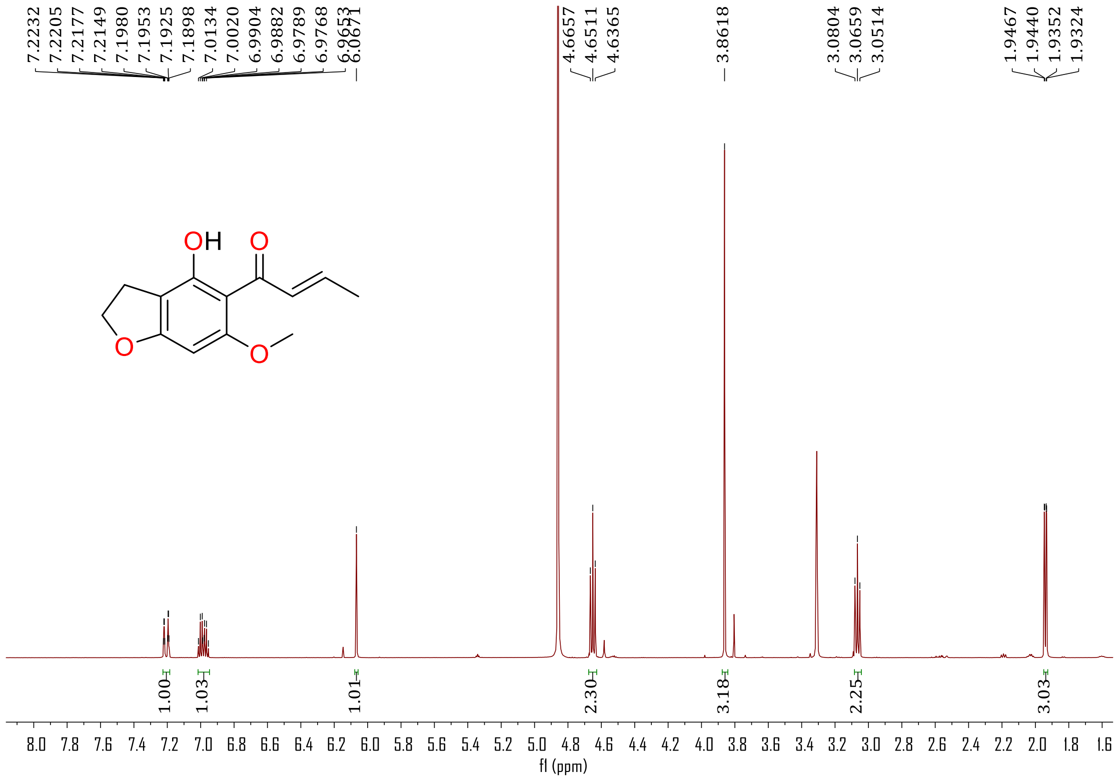

Figure S43. ^{1}H NMR spectrum (600 MHz, CD_3OD) of **5**

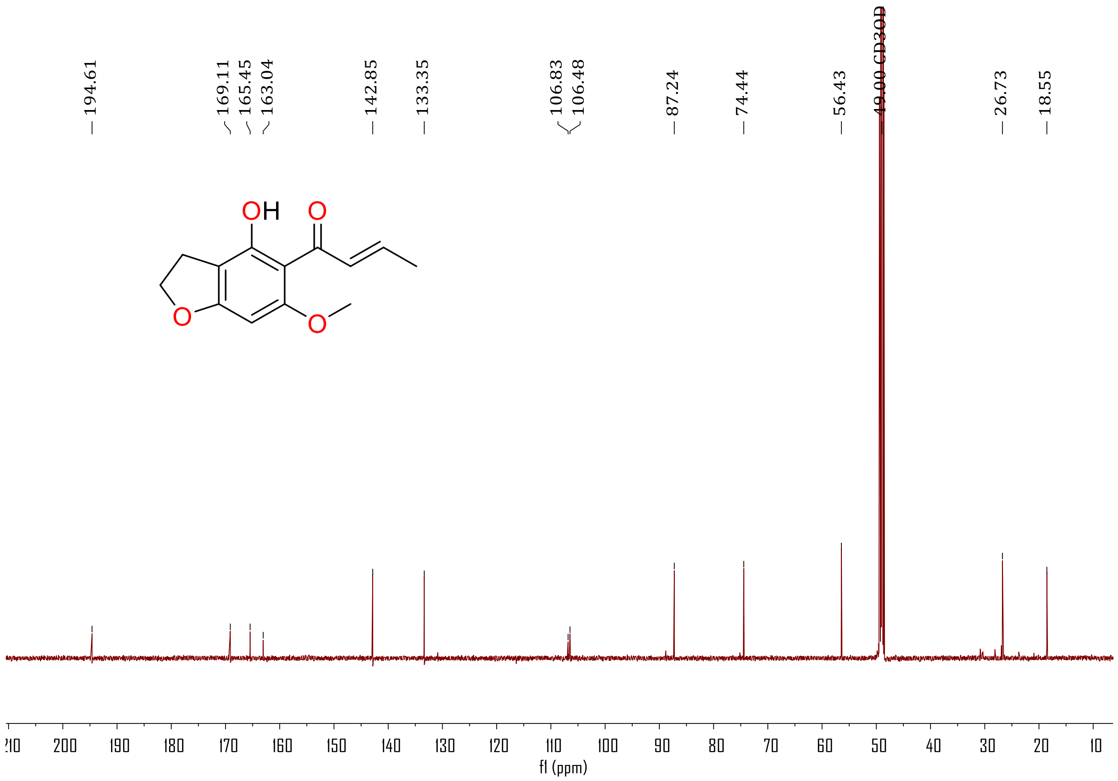

Figure S44. ^{13}C NMR spectrum (150 MHz, CD_3OD) of **5**

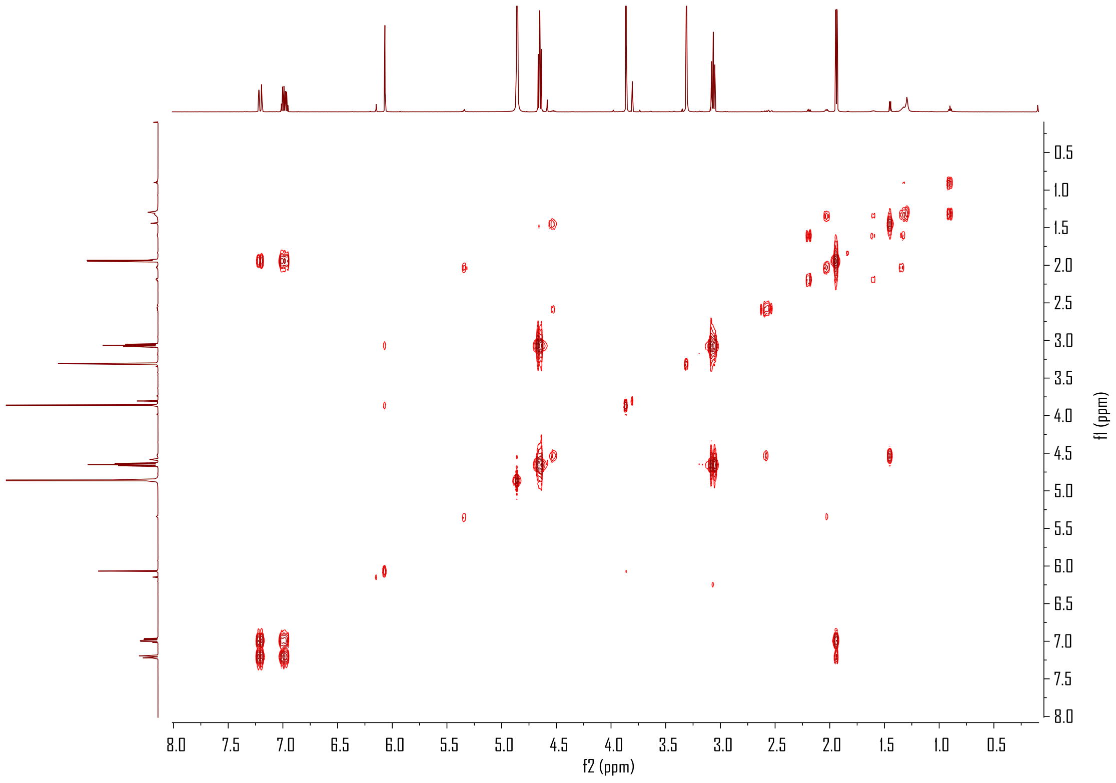

Figure S45. ^{1}H-^{1}H COSY spectrum of **5** in CD_3OD

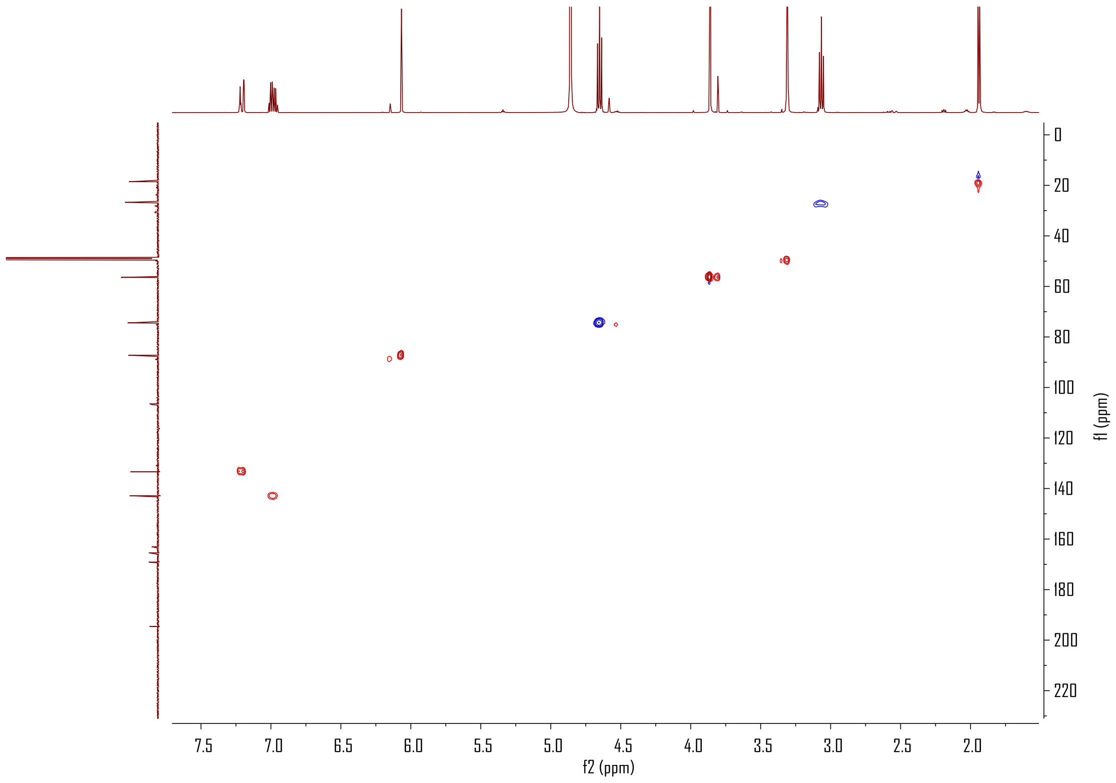

Figure S46. HSQC spectrum of **5** in CD_3OD

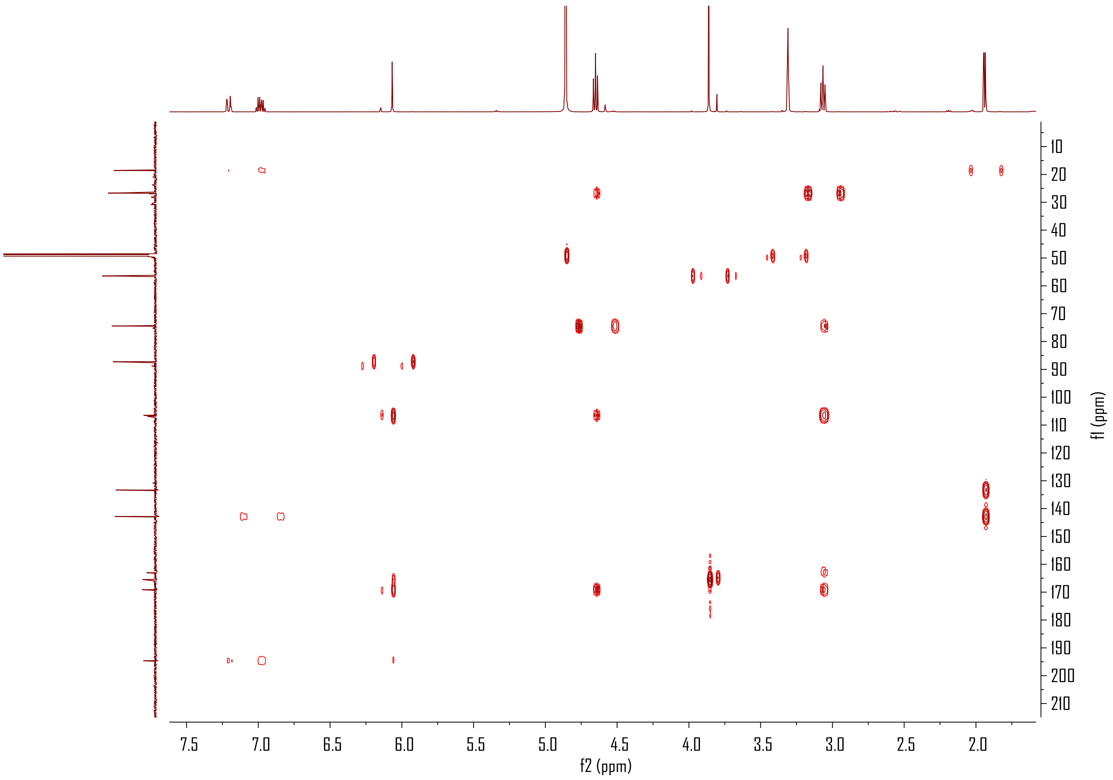

Figure S47. HMBC spectrum of **5** in CD_3OD

Mass Spectrum SmartFormula Report

Analysis Info

Analysis Name	D:\Data\MS\data\201908\chenshanchong_X 3-7_pos_42_01_7077.d	Acquisition Date	8/13/2019 3:49:47 PM
Method	LC_Direct Infusion_pos_70-500mz.m	Operator	SCSIO
Sample Name	chenshanchong_X 3-7_pos	Instrument	maXis 255552.00029
Comment			

Acquisition Parameter

Source Type	ESI	Ion Polarity	Positive	Set Nebulizer	0.4 Bar
Focus	Active	Set Capillary	4500 V	Set Dry Heater	180 °C
Scan Begin	70 m/z	Set End Plate Offset	-500 V	Set Dry Gas	4.0 l/min
Scan End	1500 m/z	Set Charging Voltage	0 V	Set Divert Valve	Waste
		Set Corona	0 nA	Set APCI Heater	0 °C

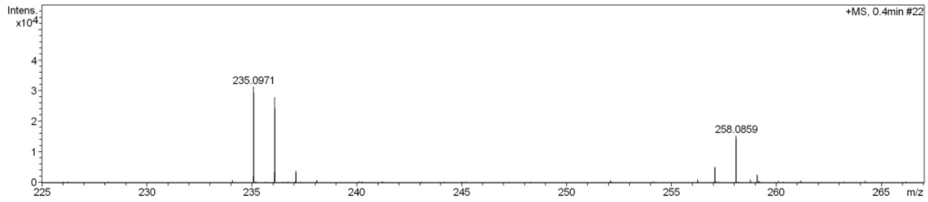

Meas. m/z	#	Ion Formula	Score	m/z	err [ppm]	err [mDa]	mSigma	rdb	e⁻ Conf	N-Rule
235.0971	1	C13H15O4	100.00	235.0965	-2.4	-0.6	432.4	6.5	even	ok
257.0783	1	C13H14NaO4	100.00	257.0784	-0.4	-0.1	632.5	6.5	even	ok

chenshanchong_X 3-7_pos_42_01_7077.d
Bruker Compass DataAnalysis 4.1 printed: 8/13/2019 3:56:30 PM by: SCSIO Page 1 of 1

Figure S48. HRESIMS spectrum of **5**

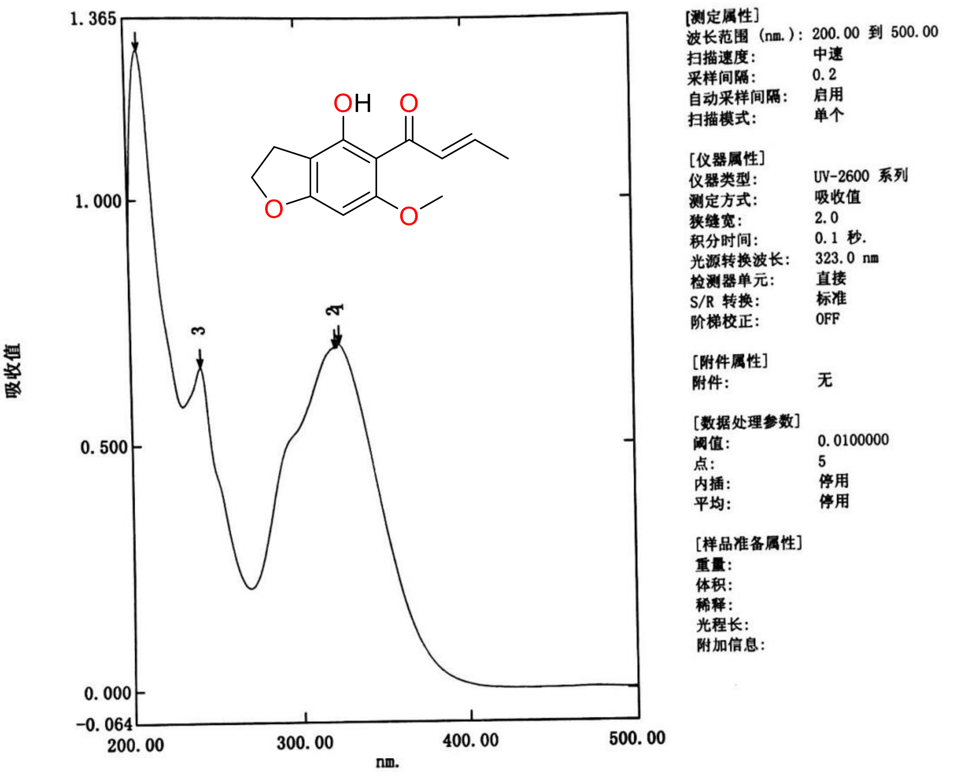

No.	P/V	波长(nm)	吸收值	描述
1	●	323.60	0.706	
2	●	321.60	0.698	
3	●	241.40	0.658	
4	●	207.20	1.300	

Figure S49. UV spectrum of **5**

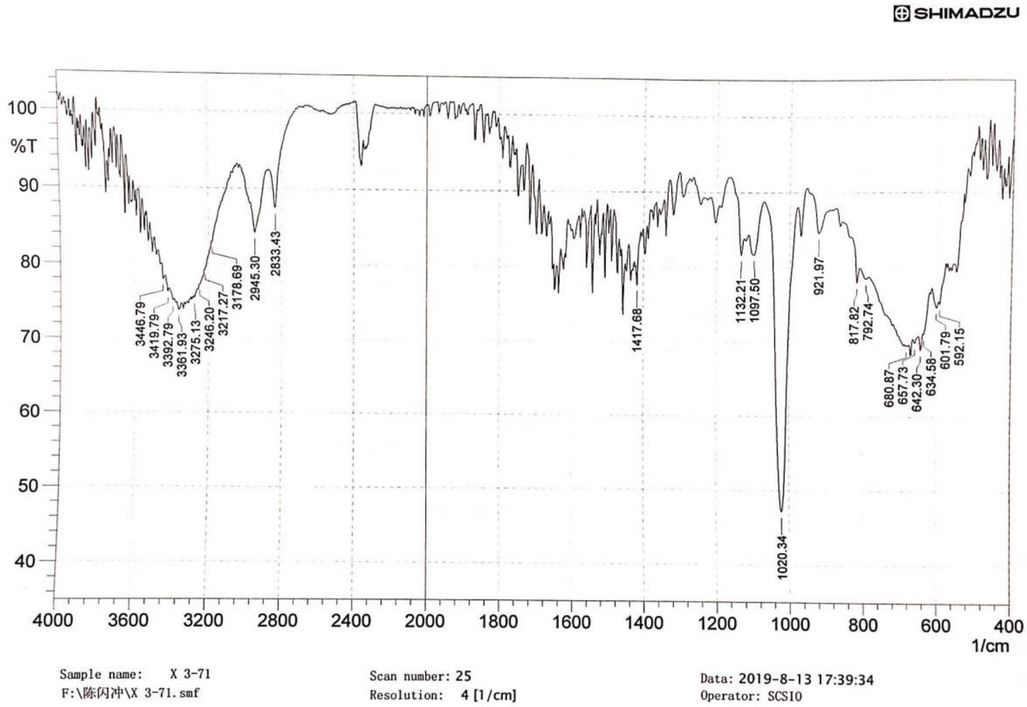

Figure S50. IR spectrum of **5**

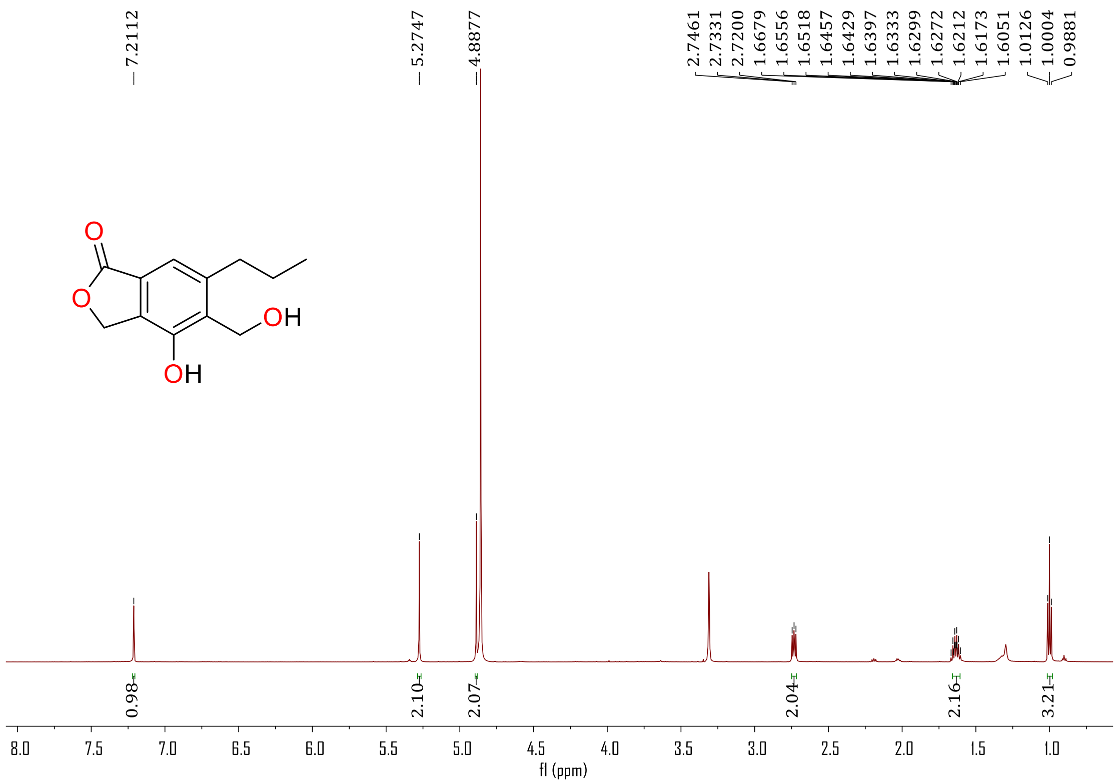

Figure S51. ^{1}H NMR spectrum (600 MHz, CD_3OD) of **6**

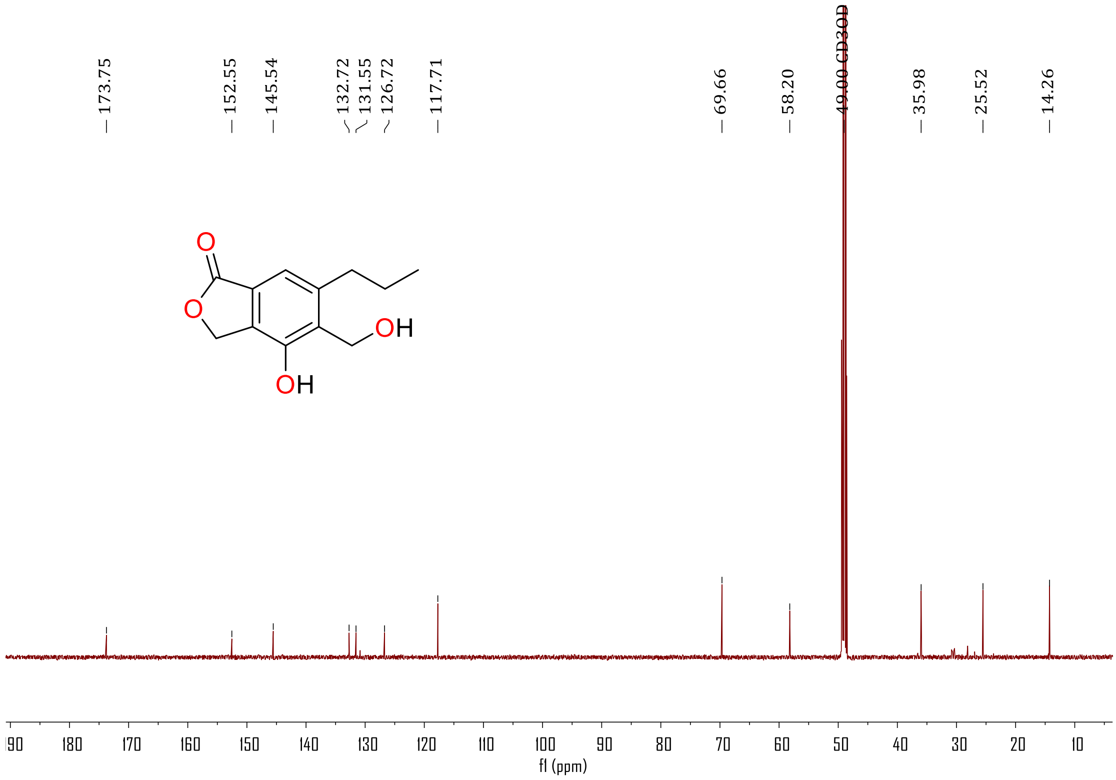

Figure S52. ^{13}C NMR spectrum (150 MHz, CD_3OD) of **6**

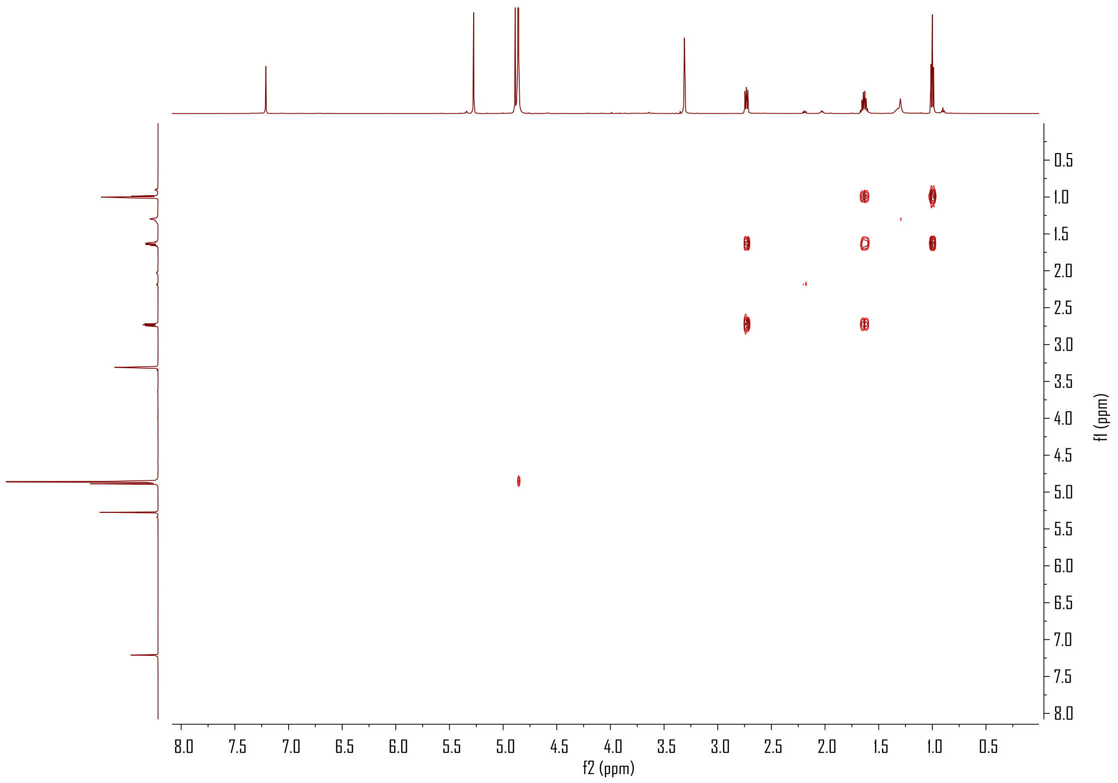

Figure S53. ^{1}H-^{1}H COSY spectrum of **6** in CD_3OD

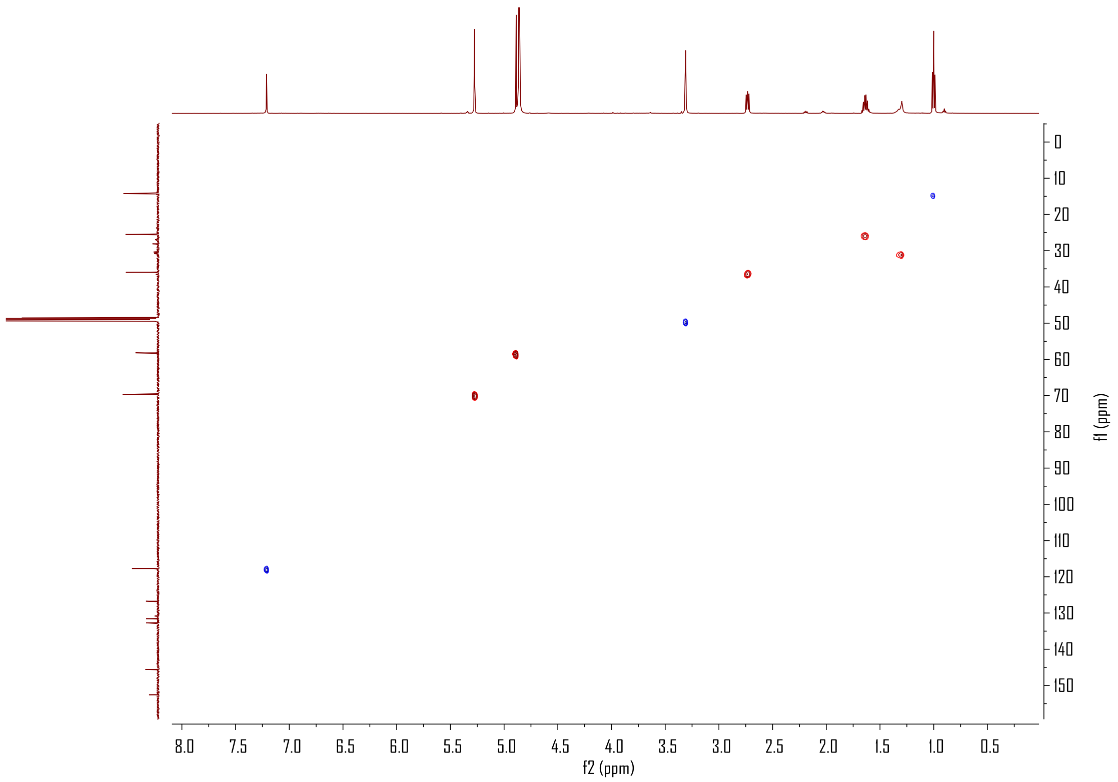

Figure S54. HSQC spectrum of **6** in CD_3OD

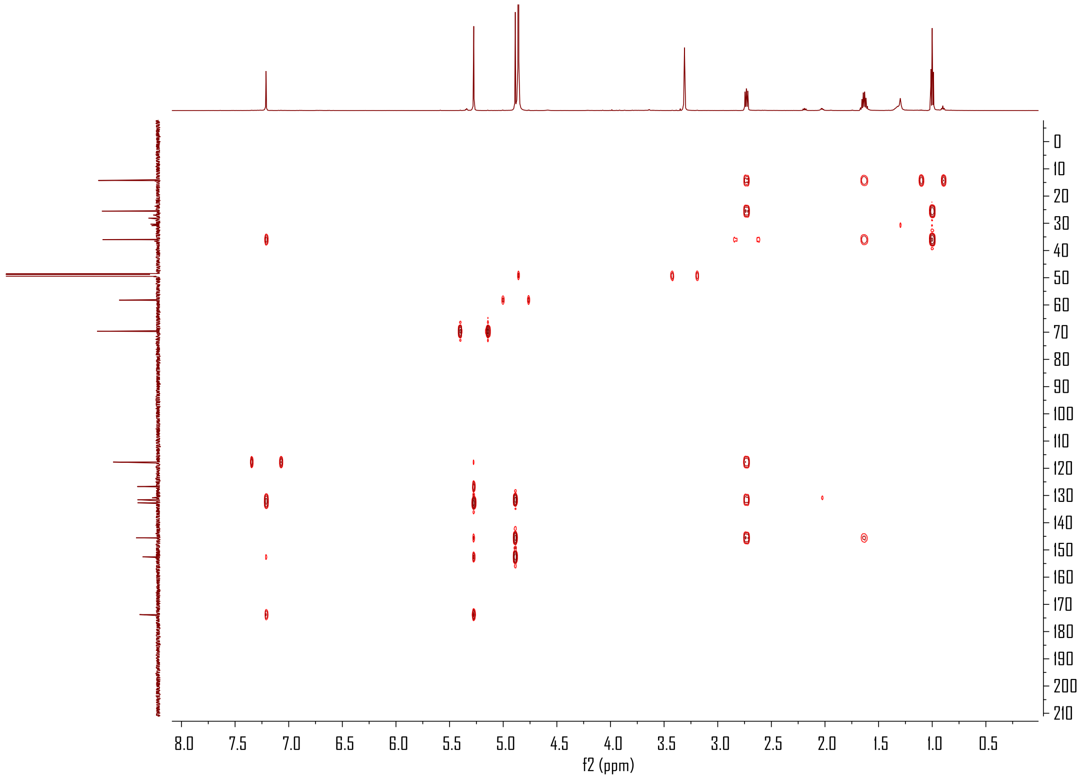

Figure S55. HMBC spectrum of **6** in CD_3OD

Mass Spectrum SmartFormula Report

Analysis Info

Analysis Name	D:\Data\MS\data\201908\chenshanchong_X 7-18_pos_43_01_7078.d	Acquisition Date	8/13/2019 3:53:15 PM	
Method	LC_Direct Infusion_pos_70-500mz.m	Operator	SCSIO	
Sample Name	chenshanchong_X 7-18_pos	Instrument	maXis	255552.00029
Comment				

Acquisition Parameter

Source Type	ESI	Ion Polarity	Positive	Set Nebulizer	0.4 Bar
Focus	Active	Set Capillary	4500 V	Set Dry Heater	180 °C
Scan Begin	70 m/z	Set End Plate Offset	-500 V	Set Dry Gas	4.0 l/min
Scan End	1500 m/z	Set Charging Voltage	0 V	Set Divert Valve	Waste
		Set Corona	0 nA	Set APCI Heater	0 °C

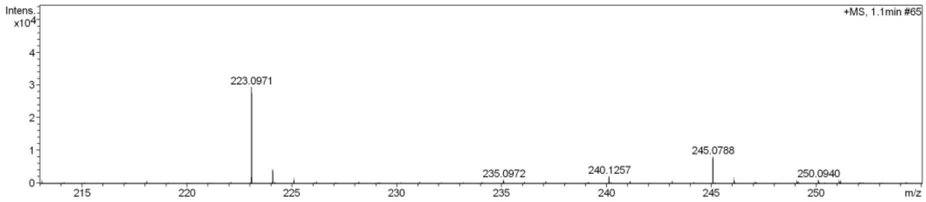

Meas. m/z	#	Ion Formula	Score	m/z	err [ppm]	err [mDa]	mSigma	rdb	e¯ Conf	N-Rule
223.0971	1	C12H15O4	100.00	223.0965	-2.8	-0.6	12.1	5.5	even	ok
245.0788	1	C12H14NaO4	100.00	245.0784	1.4	0.3	14.0	5.5	even	ok

chenshanchong_X 7-18_pos_43_01_7078.d

Bruker Compass DataAnalysis 4.1 printed: 8/13/2019 3:58:47 PM by: SCSIO Page 1 of 1

Figure S56. HRESIMS spectrum of **6**

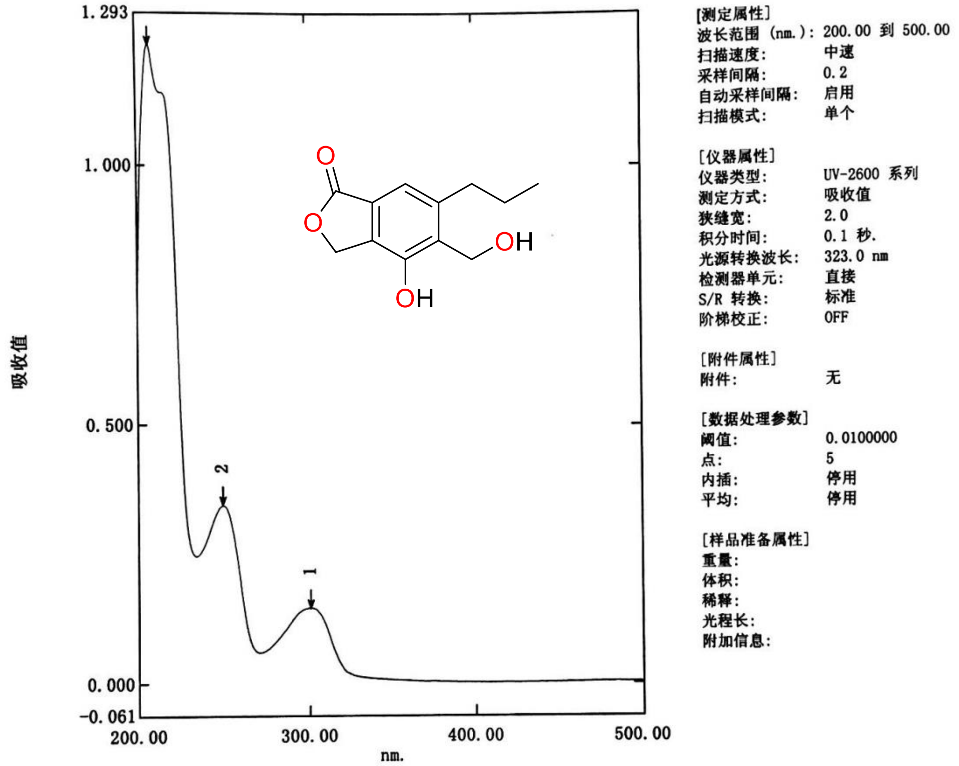

No.	P/V	波长(nm)	吸收值	描述
1	●	301.20	0.145	
2	●	249.80	0.343	
3	●	207.80	1.231	

Figure S57. UV spectrum of **6**

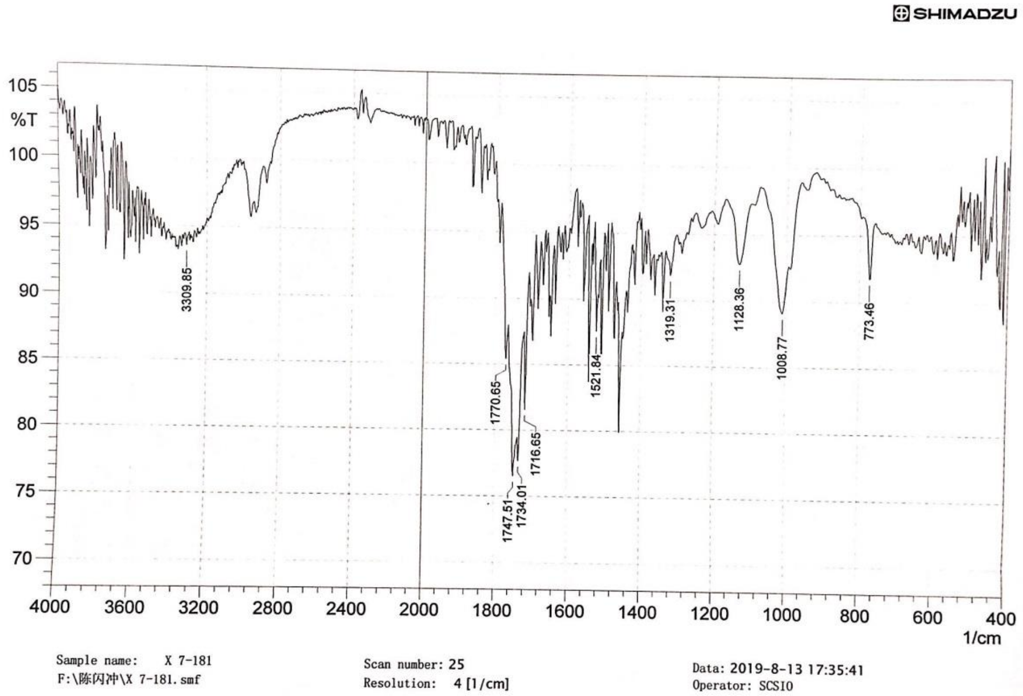

Figure S58. IR spectrum of **6**
